# Genome-wide SNP Data Reveal an Overestimation of Species Diversity in a Group of Hawkmoths

**DOI:** 10.1093/gbe/evz113

**Published:** 2019-05-29

**Authors:** Anna K Hundsdoerfer, Kyung Min Lee, Ian J Kitching, Marko Mutanen

**Affiliations:** 1 Senckenberg Natural History Collections Dresden, Dresden, Germany; 2 Ecology and Genetics Research Unit, University of Oulu, Finland; 3 Department of Life Sciences, Natural History Museum, London, United Kingdom

**Keywords:** speciation, species delineation, gene flow, RAD-sequencing, taxonomy, oversplitting

## Abstract

The interface between populations and evolving young species continues to generate much contemporary debate in systematics depending on the species concept(s) applied but which ultimately reduces to the fundamental question of “when do nondiscrete entities become distinct, mutually exclusive evolutionary units”? Species are perceived as critical biological entities, and the discovery and naming of new species is perceived by many authors as a major research aim for assessing current biodiversity before much of it becomes extinct. However, less attention is given to determining whether these names represent valid biological entities because this is perceived as both a laborious chore and an undesirable research outcome. The charismatic spurge hawkmoths (*Hyles euphorbiae* complex, HEC) offer an opportunity to study this less fashionable aspect of systematics. To elucidate this intriguing systematic challenge, we analyzed over 10,000 ddRAD single nucleotide polymorphisms from 62 individuals using coalescent-based and population genomic methodology. These genome-wide data reveal a clear overestimation of (sub)species-level diversity and demonstrate that the HEC taxonomy has been seriously oversplit. We conclude that only one valid species name should be retained for the entire HEC, namely *Hyles euphorbiae*, and we do not recognize any formal subspecies or other taxonomic subdivisions within it. Although the adoption of genetic tools has frequently revealed morphologically cryptic diversity, the converse, taxonomic oversplitting of species, is generally (and wrongly in our opinion) accepted as rare. Furthermore, taxonomic oversplitting is most likely to have taken place in intensively studied popular and charismatic organisms such as the HEC.

## Introduction

The species is a central concept of systematics and taxonomy that underpins virtually all areas of biological research, including phylogenetics, ecology, and conservation. Species delimitations are also crucial in medicine, legislation, and many other areas of human society. However, despite being such fundamental entities of nature, boundaries between species are not always well defined, and controversies are many. Typically, questions arise at the interface between populations and evolving young species. The frequent difficulties in unambiguously defining species arise because speciation is usually a slow and gradual evolutionary process during which there is a prolonged period when lineages are incompletely sorted (e.g., Maddison and Knowles 2006; Mallet et al. 2016). Additional difficulties arise from the various criteria employed in systematics to circumscribe species, that is, species concepts (De Queiroz 2007; Brunet et al. 2017). Although many species are old and are so well differentiated from other species that the boundaries between them are uncontroversial, in many other cases, unambiguous assignment of specimens into species may be extremely challenging (e.g., Martin et al. 2013; Dumas et al. 2015; Mallet et al. 2016). Although the biological reality of species has repeatedly been questioned (Lee 2003 and references therein), the need to classify biodiversity into species, even if based on pragmatic criteria, remains.

Because species are perceived as such critical biological entities, interest has increased among taxonomists into issues related to their delimitation (Zachos 2018). Most recently, molecular tools to delimit species have been developed, from single locus approaches to those based on thousands of loci (Martin et al. 2013; Pentinsaari et al. 2017). The rapid development of genomic tools has great potential to provide robust, quantitative, and standardized criteria for species delimitation (e.g., Pentinsaari et al. 2017; Dupuis et al. 2018; Luo et al. 2018; but cf., Zachos 2018). Of such tools, restriction-site associated DNA sequencing (RADseq) methods allow rapid genotyping and the accumulation of thousands of genome-wide single nucleotide polymorphisms (SNPs) (Baird et al. 2008). RADseq can be applied to nonmodel organisms for which no reference genome data are available (Baird et al. 2008; Davey and Blaxter 2010) and has been used to infer phylogenetic relationships and species trees (Chifman and Kubatko 2014; Eaton 2014) and to detect introgression and hybridization (Mallet 2005; Eaton et al. 2015; Zhang et al. 2016). Unambiguous species delimitation matching that derived from morphology, distribution, and ecology has been accomplished using RADseq data analyses of a small number of individuals in a butterfly complex (Gratton et al. 2016). In contrast, RADseq data analysis by Nieto-Montes de Oca et al. (2017) revealed substantial underestimation of species diversity in a group of lizards. Such case studies are numerous, as the discovery and naming of new species is perceived by many authors as being a major research aim. However, less attention is given to determining whether these species are actually valid biological entities because the discovery of species overestimation, and the subsequent need for taxon synonymy, is perceived as less glamorous.

The well-known spurge hawkmoths (*Hyles euphorbiae* complex, HEC; Lepidoptera: Sphingidae) offer an opportunity to study this less fashionable aspect to systematics. They are popular among both professional and amateur entomologists for the conspicuous coloration of their larvae, their large size and easy breeding, and the variability in their external patterns, colors, and mitochondrial (mt) DNA markers has been extensively studied (Hundsdoerfer et al. 2009; Hundsdoerfer, Pittaway, et al. 2011). Systematic comparison allowed the recognition of two sets of larval pattern element combinations (Hundsdoerfer, Pittaway, et al. 2011) that appear to form a north-south geographical cline in the Western Palearctic. Although the taxonomy is much debated, currently five species are recognized as valid in this region (*Hylescretica*, *H. euphorbiae*, *Hylesrobertsi*, *Hylessammuti*, and *Hylestithymali*) and there are more in Central and Eastern Asia (for a detailed explanation of the problems associated with species concepts in the HEC, see Harbich 1988). Recent studies based on mt-COI/II and nuclear EF1α sequences, as well as microsatellites, analyzed for hundreds of HEC specimens sourced from throughout Europe and adjacent Mediterranean areas, have indicated low levels of genetic variation, lack of phylogenetic resolution, and presence of mitonuclear discordance between the HEC species (Hundsdoerfer et al. 2009; Mende et al. 2016). The mitonuclear discordance is interpreted as being the result of gene flow within a broad “glacial refuge belt” and ongoing postglacial gene flow.

The present study addresses the issue of unresolved species boundaries in the HEC by applying a double-digest RADseq (ddRADseq) approach (Peterson et al. 2012) and thorough and critical data analyses. Using this genomic-scale data, we aim to 1) reassess the delimitation of the five currently valid species, 2) investigate the degree of gene flow (introgression) among the taxa of the HEC complex, and 3) amend the taxonomy and systematics to reflect the results.

## Materials and Methods

### Materials and Laboratory Techniques

**Fig. 1.**
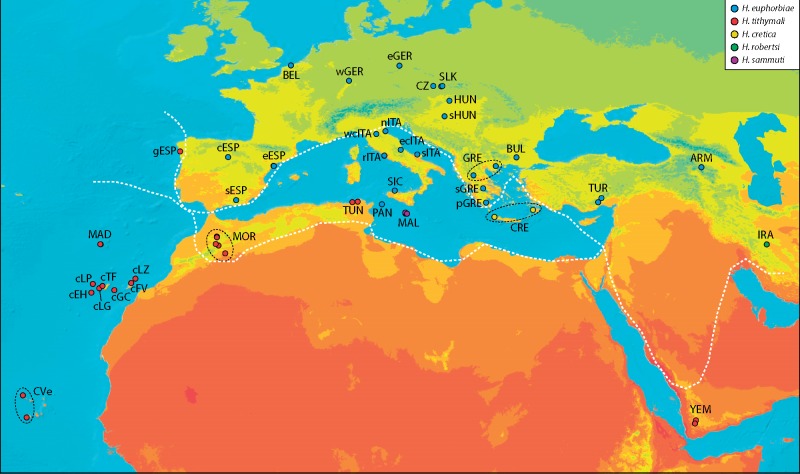
—Geographical distribution of the HEC. Geographical sampling of HEC populations mapped onto an annual mean temperature map using ArcGIS Desktop v.10.3. Temperature data were derived from WorldClim and mapped at 1-km^2^ spatial resolution with a temporal range of ∼1950–2010. Colors of circles ascribe sample sites to the five species of the currently valid taxonomy. Dashed white lines separate the two morphological species groups based on *Hyles euphorbiae* (north) and *Hyles tithymali* (south) and enclose the areas with larvae of intermediate morphology (including *Hyles cretica* and *Hyles sammuti*).

Sixty gDNA (genomic DNA) extracts from samples of the HEC (table 1 and fig. 1), plus two of *Hyles dahlii* to act as the outgroup, derived from previous studies (Hundsdoerfer et al. 2005, 2009, 2017), were chosen to generate ddRAD tag data for SNP analyses. The choice included individuals from all Western Palearctic taxa and geographic areas (six additional samples did not yield sufficient data).

**Table 1:** The Traditional taxonomy follows Kitching (2019) and includes the following taxa: *Hyles cretica* (Eitschberger, Danner and Surholt 1998); *Hyles euphorbiae conspicua* (Rothschild and Jordan 1903); *Hyles euphorbiae euphorbiae* (Linnaeus 1758); *Hyles robertsi* (Butler 1880); *Hyles sammuti* (Eitschberger, Danner and Surholt 1998); *Hyles tithymali deserticola* (Staudinger 1901); *Hyles tithymali gallaeci* (Gil-T, Requejo and Estevez 2011); *Hyles tithymali gecki* (de Freina 1991); *Hyles tithymali himyarensis* (Meerman 1988); *Hyles tithymali mauretanica* (Staudinger 1871); *H. t. mauretanica* (Staudinger 1871) x *H. t. deserticola* (Staudinger 1901); *Hyles tithymali phaelipae* (Gil-T. and Gil-Uceda 2007); and *Hyles tithymali tithymali* (Boisduval 1832)

Individual Code	Traditional Taxon	Location	Latitude	Longitude	Country	Collector	Coll. Date	ddRAD Analyzed
CRE_1254	*H. cretica*	Crete, Phalasarna	35, 51	23, 58	Greece	A. Weck-Heimann	Autumn 2005	+
CRE_4663	*H. cretica*	Dodekanes, Chalki	36, 23	27, 57	Greece	B. Gemeinholzer	2007	+
CRE_1265	*H. cretica*	Crete, Phalasarna	35, 51	23, 58	Greece	A. Weck-Heimann	Autumn 2006	+
ARM_6080	*H. e. conspicua*	Northern coast of Sevan lake, near Djil	40, 62	45, 05	Armenia	K.D. Milto	July 27, 2003	+
ARM_6082	*H. e. conspicua*	Gegharkunik, Sevan lake, Djil	40, 62	44, 95	Armenia	K.D. Milto via S. Kalyabina-Hauff	July 27, 2003	+
TUR_4327	*H. e. conspicua*	Mersin, Bolkar Daglari, Maden Köyü	37, 45	34, 63	Turkey	F. Doganlar	September 29, 2002	+
TUR_6047	*H. e. conspicua*	Mersin, Arslanköy	37, 02	34, 29	Turkey	N. Stümpel and Siegenthaler via U. Joger	July 10, 2003	+
BE_5701	*H. e. euphorbiae*	Vlaams Gewest, De Panne	51, 10	2, 58	Belgium	R. Vanovenacker	2009	+
BUL_5945	*H. e. euphorbiae*	Chaskowo, Madzharovo	41, 64	25, 85	Bulgaria	A.R. Pittaway	August–September 2009	+
BUL_5950	*H. e. euphorbiae*	Chaskowo, Madzharovo	41, 64	25, 85	Bulgaria	A.R. Pittaway	August–September 2009	+
cESP_1275	*H. e. euphorbiae*	Castilla y Léon, Roa	41, 67	−3, 91	Spain	A.K. Hundsdörfer	September 2002	+
CZ_5661	*H. e. euphorbiae*	Jihomoravsky Kraj, Bzenec	48, 98	17, 27	Czech Republic	M. Jaburek	2009	+
ecITA_6721	*H. e. euphorbiae*	Marche, Montelparo	43, 02	13, 56	Italy	M.B. Mende	August 13, 2010	
ecITA_7697	*H. e. euphorbiae*	Abruzzo, Montebello di Bertona	42, 42	13, 91	Italy	M.B. Mende	August 14, 2010	+
eESP_2587	*H. e. euphorbiae*	Platja de Malgresa, Ebro Delta	40, 77	0, 79	Spain	A.K. Hundsdörfer	September 5, 2002	+
eESP_2792	*H. e. euphorbiae*	Catalunya, Urbanització Riumar	40, 75	0, 81	Spain	A.K. Hundsdörfer	September 4, 2002	+
eGER_7563	*H. e. euphorbiae*	Sachsen, Dresdner Heide	51, 08	13, 78	Germany	M.B. Mende	July 8, 2010	+
GRE_2906	*H. e. euphorbiae*	Kendriki Makedonia, Asprovalta	40, 74	23, 72	Greece	A.K. Hundsdörfer	June 2007	+
GRE_2907	*H. e. euphorbiae*	Thessalia, Trygona	39, 79	21, 42	Greece	A.K. Hundsdörfer	June 2007	+
HUN_7578	*H. e. euphorbiae*	Pest, Budapest, Budaörs	47, 47	18, 94	Hungary	M.B. Mende	August 27, 2010	+
nCZ_8039	*H. e. euphorbiae*	Praha, Barrandov	50, 04	14, 35	Czech Republic	M.B. Mende	August 25, 2011	
nITA_7663	*H. e. euphorbiae*	Emilia-Romagna, Lido di Classe	44, 33	12, 33	Italy	M.B. Mende	August 13, 2010	+
PAN_7658	*H. e. euphorbiae*	Pantelleria, Rukia, airport	36, 81	11, 97	Italy	A. Corso	May 6, 2010	+
pGRE_2493	*H. e. euphorbiae*	Peloponnes, Geronthri, Geraki	36, 98	22, 72	Greece	A.K. Hundsdörfer	April 2007	+
rITA_5640	*H. e. euphorbiae*	Lazio, Roma airport, Focene	41, 81	12, 22	Italy	M.B. Mende	September 21, 2009	+
rITA_5646	*H. e. euphorbiae*	Lazio, Roma airport, Fregene	41, 83	12, 23	Italy	M.B. Mende	September 21, 2009	+
sESP_2402	*H. e. euphorbiae*	Andalucía, Alcudia de Guadix	37, 24	−3, 08	Spain	A.K. Hundsdörfer	May 2003	+
sGRE_2491	*H. e. euphorbiae*	Sterea Ellada, Itea	38, 45	22, 42	Greece	A.K. Hundsdörfer	April 2007	+
sHUN_6707	*H. e. euphorbiae*	Hungary, Baranya, Villány	45, 86	18, 43	Hungary	M. Vamberger and U. Fritz	June 4, 2011	+
SIC_7587	*H. e. euphorbiae*	Sicilia, Sferracavallo	38, 21	13, 29	Italy	M.B. Mende	May 13, 2010	+
SIC_7589	*H. e. euphorbiae*	Sicilia, Sferracavallo	38, 21	13, 29	Italy	M.B. Mende	May 13, 2010	+
sITA_6450	*H. e. euphorbiae*	Puglia, Gargano Blu	41, 93	15, 62	Italy	A.K. Hundsdörfer	September–October 2004	+
SLK_5680	*H. e. euphorbiae*	Trenciansky Kraj, Nemsova	48, 96	18, 12	Slovakia	J. Macko	2009	+
SLK_5684	*H. e. euphorbiae*	Trenciansky Kraj, Trenciansca Tepla	48, 94	18, 10	Slovakia	J. Macko	2009	+
SLK_5694	*H. e. euphorbiae*	Trenciansky Kraj, Dubnica nad Vahom	48, 98	18, 21	Slovakia	J. Macko	2009	+
wcITA_7709	*H. e. euphorbiae*	Toscana, Firenze, Passo di Giogo	44, 05	11, 39	Italy	M.B. Mende	August 16, 2010	+
wGER_8926	*H. e. euphorbiae*	Hessen, Viernheim	49, 53	8, 57	Germany	A.K. Hundsdörfer	August 21, 2001	+
IRA_4231	*H. robertsi*	Esfahan, Esfahan	32, 65	51, 68	Iran	A.R. Pittaway	June 2001	
IRA_4243	*H. robertsi*	Esfahan, Esfahan	32, 65	51, 68	Iran	A.R. Pittaway	June 2001	+
IRA_5564	*H. robertsi*	Esfahan, Natanz, Karkas Mts.	33, 45	51, 88	Iran	A. Naderi	May 21–24, 2009	
IRA_5565	*H. robertsi*	Esfahan, Natanz, Karkas Mts.	33, 45	51, 88	Iran	A. Naderi	May 21–24, 2009	
MAL_3133	*H. sammuti*	Malta, Dingli Cliffs	35, 85	14, 40	Malta	A.K. Hundsdörfer	October 2007	+
MAL_3190	*H. sammuti*	Malta, Mellieha, Fort Campbell	35, 96	14, 39	Malta	A.K. Hundsdörfer	October 2007	+
MAL_5899	*H. sammuti*	Malta, Marsaxlokk	35, 84	14, 56	Malta	A.K. Hundsdörfer	October 2007	+
MOR_5356	*H. t. deserticola*	Meknès-Tafilalet, Errachidia	31, 74	−4, 20	Morocco	A.K. Hundsdörfer	May 25, 2003	+
gESP_8626	*H. t. gallaeci*	Galicia, Pontevedra, Donón, dunas de Barra	42, 26	−8, 85	Spain	F. Gil-T.	May 2011	+
MAD_6086	*H. t. gecki*	Madeira, Ribeira Brava/Funchal	32, 67	−17, 06	Portugal	S. Kalyabina-Hauff	January 15, 2003	+
MAD_6088	*H. t. gecki*	Madeira, Ponta do Sol	32, 68	−17, 10	Portugal	S. Kalyabina-Hauff	January 22, 2003	+
YEM_8748	*H. t. himyarensis*	Dhamar, Dhamar	14, 53	44, 38	Yemen	C. Naumann	2001/2002	+
YEM_8936	*H. t. himyarensis*	Ibb, Naqil (Sumarah Pass)	14, 20	44, 28	Yemen	C. Naumann	June 2001	+
MOR_1382	*H. t. mauretanica*	Meknès-Tafilalet, Ifrane	33, 49	−5, 07	Morocco	A.K. Hundsdörfer	May 24, 2003	+
MOR_6093	*H. t. mauretanica*	Meknès-Tafilalet, Midelt, Taouraout (near Cirque de Jaffar)	32, 55	−4, 90	Morocco	O. Niehuis	June 27, 2002	+
MOR_6100	*H. t. mauretanica*	Meknès-Tafilalet, Azrou, Michlifene	33, 40	−5, 07	Morocco	O. Niehuis	June 28, 2002	+
MOR_6094	*H. t. mauretanica*	Meknès-Tafilalet, Boumia, Sidi Tiar	32, 70	−5, 18	Morocco	O. Niehuis	June 25, 2002	+
TUN_1839	*H. t. mauretanica*	Near Ghezala	37, 08	9, 48	Tunisia	A.K. Hundsdörfer	October 21, 2004	+
TUN_1835	*H. t. mauretanica* x *deserticola*	Jendouba, Tabarka	37, 03	8, 91	Tunisia	A.K. Hundsdörfer	September–October 2004	+
cLP_1650	*H. t. phaelipae*	Islas Canarias, La Palma	28, 57	−17, 87	Spain	A.K. Hundsdörfer	March–April 2002	+
cEH_1499	*H. t. phaelipae*	Islas Canarias, El Hierro	27, 68	−18, 02	Spain	A.K. Hundsdörfer	March–April 2002	+
cFV_1784	*Hyles t. tithymali*	Islas Canarias, Fuerteventura	28, 67	−13, 91	Spain	A.K. Hundsdörfer	March–April 2002	+
cGC_1450	*Hyles t. tithymali*	Islas Canarias, Gran Canaria	27, 93	−15, 65	Spain	A.K. Hundsdörfer	March–April 2002	+
cLG_1554	*Hyles t. tithymali*	Islas Canarias, La Gomera	28, 12	−17, 20	Spain	A.K. Hundsdörfer	March–April 2002	+
cLZ_1624	*Hyles t. tithymali*	Islas Canarias, Lanzarote	29, 11	−13, 47	Spain	A.K. Hundsdörfer	March–April 2002	+
cTF_4822	*Hyles t. tithymali*	Islas Canarias, Teneriffa	28, 36	−16, 87	Spain	A.K. Hundsdörfer	March–April 2002	+
CVe_1892	*Hyles t. tithymali*	Brava, Nova Sintra	14, 83	−24, 70	Cabo Verde	E. Aistleitner	November 3, 2005	+
CVe_1904	*Hyles t. tithymali*	Fogo, Bordeira, SW via Mosteiros	14, 91	−24, 39	Cabo Verde	E. Aistleitner	November 30, 2005	
CVe_7527	*Hyles t. tithymali*	Santo Antao, Espongeiro	17, 10	−25, 09	Cabo Verde	J. Batelka and J. Straka	October 16, 2009	+

Note.—Samples included in the final ddRAD data sets are marked in the right column.

The quantity and quality of gDNA extracts were checked using PicoGreen (Molecular Probes) and a NanoDrop (Thermo Scientific) for the ddRADseq library preparation. To reach sufficient gDNA quantity and quality, whole genome amplification was performed due to low concentrations of gDNA in the original extracts (comparable numbers of tags were expected, because the coverage of the genome can be expected to be the same regardless whether native or amplified DNA is used, Cruaud et al. 2018). The ddRAD library was implemented following the protocols described in Lee et al. (2018) with two exceptions: gDNA was digested with *Pst*I and *Msp*I and the size distribution was measured with Bioanalyzer (Agilen Technologies). The demultiplexed fastq data are archived in the NCBI SRA (BioProject ID: PRJNA352456).

### Assembly, SNP Calling, Phylogenetic, and Coalescent Analyses

We processed raw Illumina reads using pyRAD v.3.0.64 (Eaton 2014) and *ipyrad* (Eaton and Overcast 2016) pipelines, and allowed for the inclusion of indel variation, which might contain divergent individuals, using global alignment clustering for phylogenetic studies. We demultiplexed samples using their unique barcode and adapter sequences. Sites with Phred quality scores below 20 were converted to “*N*” characters and reads with ≥10% *N*’s were discarded. Sequences were clustered using the *vsearch* program (http://github.com/torognes/vsearch; Last accessed 29 March 2019), and then aligned using MUSCLE (Edgar 2004). The *vsearch* clustering step establishes homology among reads within a sample. As an additional filtering step, consensus sequences that had low coverage (<3 reads), excessive undetermined or heterozygous sites (>3), or too many haplotypes (>2 for diploids) were discarded. The consensus sequences were clustered across samples at 80%, 85%, and 90% similarity (clustering threshold, *c*). This step establishes locus homology among individuals. A locus represents a filtered and aligned read that passed the pyRAD parameters given above; it has also been used as a tag or stack in RAD-sequencing data. Each locus was aligned using MUSCLE, and a filter applied to detect potential paralogs, as a shared heterozygous site across many samples likely represents clustering of paralogs with a fixed difference rather than a true heterozygous site. We applied a filter that allowed a maximum proportion of shared polymorphic sites at a given locus (*p* = 30%).

Different clustering thresholds (*c*80%, 85%, and 90%) and the minimum taxon coverage at a given locus (*m*) had a large effect on the number of loci, SNPs, variable sites and parsimony-informative sites (supplementary table S1, Supplementary Material online). The final loci to be analyzed were chosen by adjusting the value of *m* (supplementary table S1, Supplementary Material online), which specifies the minimum number of individuals that are required to have data at a locus for that locus to be included in the final matrix. We compiled data matrices with *m* values of 6, 10, 15, 20, 25, and 30 to explore the potential effects of loci, SNPs, variable sites, parsimony-informative sites, and proportion of missing data on phylogenetic analyses.

Pairwise sequence divergences based on K2P distances and pairwise *F*_ST_ values were calculated from the “HEC_c85m6” data matrix (i.e., a clustering threshold of 85% similarity and an *m* value of 6, requiring that a locus contain data for at least six samples; see supplementary table S1, Supplementary Material online) using MEGA6 and Arlequin v.3.5 (Excoffier and Lischer 2010; Tamura et al. 2013). Statistical significance of the *F*_ST_ values was tested by permutation analysis with 1,000 permutations. The proportion of missing data was calculated using Mesquite (Maddison and Maddison 2017).

We used a maximum likelihood approach implemented in RAxML v.8.2.0 (Stamatakis 2014) for phylogenetic analyses because of its ability to handle very large data sets efficiently. We used the concatenated sequences from all recovered RAD loci from the “HEC_c85m6” matrix with a GTR+GAMMA model of sequence evolution and bootstrap support estimated using a 500 replicate rapid-bootstrap analysis. We visualized the resulting phylogeny and assessed bootstrap support using FigTree v.1.4.2 (Rambaut 2015). In addition, a coalescent SVDquartets analysis was conducted in PAUP 4.0a163 (https://paup.phylosolutions.com/; Last accessed 25 February 2019) on the concatenated RADseq locus sequence data of the “HEC_c85m30” data matrix, supplementary table S1, Supplementary Material online), with the five currently valid species set as taxon partitions. We used default settings to yield both a lineage and a species tree and ran 1,000 bootstrap replicates, on both lineage and species levels. The two trees were formatted using FigTree v.1.4.2 (Rambaut 2015) and combined using Adobe Illustrator (vCS2) to show the lineages branching within the species tree.

### Population Genomic Analysis

We inferred population clustering with admixture from SNP frequency data to better visualize genomic variation between individuals with Structure v.2.3.1 (Pritchard et al. 2000). We used 10,093 putatively unlinked SNPs, sampled by selecting a single SNP from each locus in the “HEC_c85m6” data matrix (supplementary table S1, Supplementary Material online). Ten replicates were run with each value of *K*, defined as populations or genetic groups assumed, between 1 and 5. Each run had a burn-in of 50K generations followed by 500K generations of sampling. Replicates were permuted using CLUMPP (Jakobsson and Rosenberg 2007) and the optimal *K* value inferred using Structure Harvester (Earl and VonHoldt 2012) according to the ad hoc Δ*K* statistic (Evanno et al. 2005), which is the second-order rate of change of the likelihood function. Structure results were visualized using DISTRUCT (Rosenberg 2004).

We used SplitsTree v.4.14.2 (Huson and Bryant 2006) to construct a phylogenetic network from the “HEC_c85m30” data matrix, implementing the Neighbor-net (ordinary least squares variance) and equal angle algorithms, using uncorrected *p*-distances with heterozygous ambiguities averaged and normalized, and 1,000 bootstrap replicates (>75% shown), as well as a maximum parsimony analysis.

Isolation by distance (IBD) was tested, based on the pairwise genetic distance (the “HEC_c85m30” data matrix) and geographical distance, using IBD v. 1.52 (Bohonak 2002). Geographical distance was estimated as the straight-line distance between the GPS coordinates of the sampled populations using the Geographic Distance Matrix Generator (Ersts 2018). To assess statistical correlation among matrices, we applied Mantel tests (1,000 randomizations) to distance matrices.

We also explored variation among the HEC samples at the retained putative ddRAD loci (“HEC_c85m6” data matrix) by performing a principal components analysis (PCA) using the *dudi.pca* R function in the “*ade4*” package (Dray and Dufour 2007).

### Admixture Tests

We used four-taxon *D*-statistic for introgression analysis (Durand et al. 2011). In our study, we were interested in testing whether introgression had occurred among *H. cretica*, *H. euphorbiae*, *H. robertsi*, *H. sammuti*, and *H. tithymali*, because mitochondrial and nuclear sequences, and microsatellites from those species were either mixed or genetically very closely related (Hundsdoerfer et al. 2009; Mende and Hundsdoerfer 2013; Mende et al. 2016). All loci from the HEC_c85m6 data set were used in the *D*-statistic tests, and heterozygous sites were included in the analyses. We had multiple individuals of each of the five currently valid species except *H. robertsi* (due to failure to obtain RADseq data), and *D*-statistic tests were performed using all possible combinations between individuals from the five HEC species. Two individuals of *H. dahlii* were used as outgroup (O). In total, 80 tests were conducted, and for each test 1,000 bootstrap replicates were performed to measure the standard deviation of the *D*-statistic. Significance was evaluated by converting the *Z*-score (which represents the number of standard deviations from zero for *D*-statistic) into a two-tailed *P* value, using *α* = 0.01 as a conservative cutoff for significance after correcting for multiple comparisons using Holm–Bonferroni correction. All *D*-statistics were measured in pyRAD v.3.0.64 (Eaton 2014). To run interactive data analysis, Python Jupyter notebooks (http://jupyter.org; Last accessed 16 April 2019) were used.

### Genome-wide Bayesian Species Delimitation of the HEC Group

We performed Bayes factor (BF) species delimitation using the BFD* method (Leaché et al. 2014), as implemented using the SNAPP (Bryant et al. 2012) plugin for BEAST2 v.2.0.2 (Bouckaert et al. 2014), which allows for the comparison of alternative species delimitation models in an explicit multispecies coalescent framework using genome-wide SNP data (“HEC_c85m30” data matrix). Because it was not computationally feasible to test all possible scenarios, we selected and tested 19 competing species models (see supplementary table S2, Supplementary Material online) according to the currently valid taxonomy. For all tested models, we conducted path sampling for a total of 24 steps (100,000 Markov chain Monte Carlo steps and 10,000 burn-in steps each) to calculate the marginal likelihood estimation (MLE) for each competing model. We tested 19 competing species models: 1) “**Lump all**”: treating all five traditionally accepted species together as a single unit; 2) “**2 sp_ce**”: two species model composed of (*cretica* + *euphorbiae*) and (*robertsi* + *sammuti* + *tithymali*); 3) “**2 sp_cr**”: (*cretica* + *robertsi*) and (*euphorbiae* + *sammuti* + *tithymali*); 4) “**2 sp_cs**”: (*cretica* + *sammuti*) and (*euphorbiae* + *robertsi* + *tithymali*); 5) “**2 sp_ct**”: (*cretica* + *tithymali*) and (*euphorbiae* + *robertsi* + *sammuti*); 6) “**2 sp_er**”: (*euphorbiae* + *robertsi*) and (*cretica* + *sammuti* + *tithymali*); 7) “**2 sp_es**”: (*euphorbiae* + *sammuti*) and (*cretica* + *robertsi* + *tithymali*); 8) “**2 sp_et**”: (*euphorbiae* + *tithymali*) and (*cretica* + *robertsi* + *sammuti*); 9) “**2 sp_rs**”: (*robertsi* + *sammuti*) and (*cretica* + *euphorbiae* + *tithymali*); 10) “**2 sp_rt**”: (*robertsi* + *tithymali*) and (*cretica* + *euphorbiae* + *sammuti*); 11) “**2 sp_st**”: (*sammuti* + *tithymali*) and (*cretica* + *euphorbiae* + *robertsi*); 12) “**2 sp_c**”: “Lump all” model plus a split of *cretica*; 13) “**2 sp_e**”: “Lump all” model plus a split of *euphorbiae*; 14) “**2 sp_r**”: “Lump all” model plus a split of *robertsi*; 15) “**2 sp_s**”: “Lump all” model plus a split of *sammuti*; 16) “**2 sp_t**”: “Lump all” model plus a split of *tithymali*; 17) “**3 sp_cer**”: three species model that contains (*cretica* + *euphorbiae* + *robertsi*), *sammuti*, and *tithymali*; 18) “**4 sp_ce**”: four species model containing (*cretica* + *euphorbiae*), *robertsi*, *sammuti*, and *tithymali*; and 19) “**Trad taxon**”: traditional taxonomy based on morphological descriptions.

BF support was compared among models to identify the best-supported species model. We visualized the best-supported BF species tree posterior from the final path sampling step (minus a 10% burn-in) using DensiTree v.2.2.1 (Bouckaert 2010) for comparison with the SVDquartets species tree.

## Results

### Optimization of RAD Loci Parameters

The ddRADseq analysis of the 62 individuals of the HEC (supplementary table S4, Supplementary Material online) yielded 250,000 reads per individual on average, of which 73.3% were retained after filtering. We selected the parameter combination HEC_*c*85*m*6 (supplementary table S1, Supplementary Material online) for phylogenetic analysis as it had the optimal combination of loci-clustering parameters for the HEC in that it maximized the fraction of variable sites that were phylogenetically informative. The data comprised a total of 2,174,137 bp, including 11,276 loci and 10,093 SNPs shared across more than at least 6 individuals at a given locus (HEC_c85m6; supplementary table S1, Supplementary Material online), and included 114,463 variable sites, of which 56,033 were parsimony informative. Pairwise divergence among the 60 HEC individuals ranged up to 2.25%.

### HEC Cluster Formation, Coalescence, Introgression, and Gene Flow

**Fig. 2.**
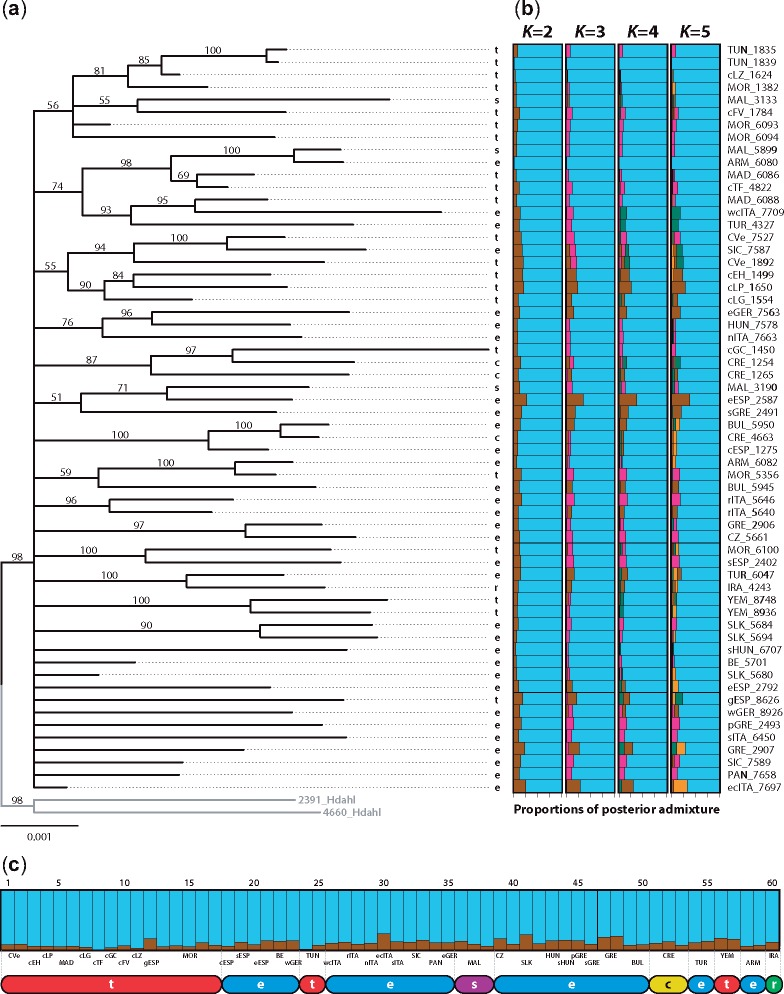
—Phylogenetic hypothesis and HEC population structuring. Phylogenetic relationships and population structuring of the HEC including two specimens of *Hyles dahlii* (HEC s.l.) as outgroup based on the *c*85*m*6 data matrix, which consisted of 10,093 unlinked SNPs in 2,174,137 bp. (*a*) ML tree inferred from RAxML analysis. The maximal internal distance of 2% within the HEC is found between ecITA_7697 (Italy) and SLK_5684 (Slovakia). Branch support was inferred with 500 bootstrap replicates; bootstrap values are indicated above each branch. (*b*) Admixture graphs of *K* = 2–5 source populations. The blue color was chosen to refer to *Hyles euphorbiae*, the other four colors (brown, orange, dark green, and pink) distinguish four further possible groups but have not been coded to refer to taxonomy or phylogeny. (*c*) Individual cluster assignment probabilities for the *K* = 2 ordered by geographical distribution. Population abbreviations correspond to figure 1. Abbreviations refer to species definitions according to currently valid taxonomy: c, *Hyles cretica*; e, *H. euphorbiae*; r, *Hyles robertsi*; s, *Hyles sammuti*; and t, *Hyles tithymali*. The colors coding this taxonomy correspond to those in figure 1.

The polytomy at the base of the phylogenetic tree (fig. 2*a*) reflects an evidentiary lack of differentiation within the HEC at the genomic level. Although the entire HEC is well supported (98% bootstrap), no internal species clusters are formed, neither the two species with only three samples each (*H. sammuti* and *H. cretica*) nor the two well-sampled species, *H. euphorbiae* and *H. tithymali*. Of the 60 specimens included in the phylogenetic analysis (fig. 2*a*), 12 do not group with any others, 27 form 11 small clusters of 2–3 specimens (of which only five have a support >97%) and the remaining 21 samples form 3 groups of 6, 7, and 8 specimens, respectively (all three with low support values of 55–54%). Only 20 specimens (one third of the ingroup) form subgroups supported by more than 97% bootstrap support. Ten pairs of samples form fully supported clusters but three of these group together individuals that would be traditionally determined as two different species and are from localities that are hundreds of kilometers apart (*H. sammuti* #5859 from Malta and *H. euphorbiae* #6080 from Armenia; *H. tithymali* #7527from Cape Verde Islands and *H. euphorbiae* #7587 from Sicily; and *H. tithymali* #5356 from Morocco and *H. euphorbiae* #6082 from Armenia). Most samples on the tree do not group according to either country of origin or traditional species definition. Additionally, the Mantel tests showed no significant correlation between genetic differentiation and geographical distance (*r*_M_ = −0.001734, *P *>* *0.494; supplementary fig. S1, Supplementary Material online).

Every single HEC individual is assigned to the blue group with over 80% admixture by Structure (fig. 2*b*). Population clustering analyses (fig. 2*b* and *c*) sorted the individuals into two groups (*K *=* *2, Δ*K *=* *8.20; see supplementary table S5, Supplementary Material online) according to the ad hoc Δ*K*, every single HEC individual is assigned to the blue group at over 80% admixture. The second, red group shows only a varying, but comparatively low level of admixture (<20%), with the highest values on the European mainland (fig. 2*c*). The five traditionally delimited HEC species are indistinguishable under all values of *K* (the number of distinct clusters; fig. 2*b*). Although the Evanno method could not achieve a result for *K *=* *1, the lack of any internal structure very clearly demonstrates that all HEC specimens belong to a single group only, in line with the phylogenetic analyses (fig. 2*a*).

*F*_ST_ values revealed *H. robertsi* as the most divergent species (supplementary fig. S2, Supplementary Material online), a genetic differentiation between *H. robertsi* and *H. euphorbiae* of up to 39.5% is unexpected. However, this is probably an artifact in the calculation of allele frequency covariance over variable sites due to the high proportion of missing data and having only a single individual for that species (due to failure to obtain RADseq data). *Hyles cretica*, *H. sammuti*, *H. euphorbiae*, and *H. tithymali* were only weakly differentiated; *F*_ST_ values were low; ranging from 0.006 to 0.116. Permutation tests for genetic differentiation among the species suggest random assemblages of individuals (no values were significant), demonstrating that overall the HEC is panmictic.

Both SplitsTree network analyses show weak differentiation of the HEC (supplementary fig. S3, Supplementary Material online). The starlike topologies again illustrate the lack of subclades in the HEC. Furthermore, internal box formation indicates uncertainty in clade formation.

Across all the phylogenetic, cluster and network analyses, there is no discernible genomic differentiation among the HEC species. Although the first component of the PCA shows a weak genomic differentiation into two groups, the three species, *H. cretica*, *H. robertsi*, and *H. sammuti* merged into either *H. euphorbiae* or *H. tithymali* (supplementary fig. S4, Supplementary Material online).

**Fig. 3.**
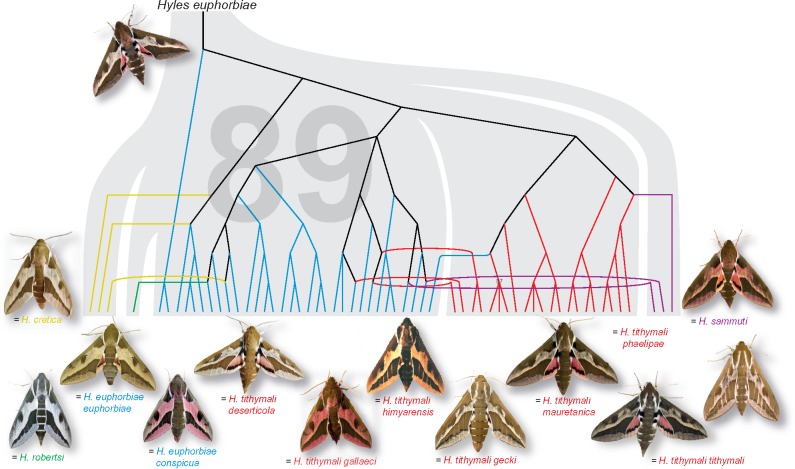
—SVDquartets coalescence analyses. The broad gray branches show the SVDquartets species tree according to valid taxonomy based on the *c*85*m*30 sequence data matrix, which consisted of 835 unlinked SNPs in 160,778 bp (outgroup *Hyles dahlii* pruned). We overlaid the lineage tree and positioned the individuals according to species affiliation by extending their branches by hand to illustrate gene flow. Bootstrap support values above 80% are shown at nodes (there are only two: one value 89 in the species tree in large font size supporting the crown group of *Hyles cretica*, *Hyles robertsi*, and *Hyles euphorbiae*, and one value 87 in the lineages tree in smaller font size). The taxonomy color code corresponds to that in figure 1.

Although the SVDquartets coalescent lineage analysis (fig. 3; outgroup pruned) provides more resolution than the RAxML tree, the samples of the five species as currently defined do not form clades there either. The SVDquartets species tree shows a different topology to that of the samples in the lineage tree, in that *H. sammuti* branches off first, followed by *H. tithymali*. The only well supported clade in the species tree (bootstrap support of 89%) is that of *H. cretica* plus the clade comprising *H. euphorbiae* plus the single *H. robertsi*. However, *H. cretica* consists only of individuals that arise from *H. euphorbiae* branches, and *H. sammuti* of individuals that arise from both *H. euphorbiae* and *H. tithymali* in the lineage tree. Overlaying the lineage tree onto the species tree allows comparison of the results. On the lineage level, the *H. euphorbiae* samples, plus *H. robertsi* and *H. cretica*, form the paraphyletic base and *H. tithymali* the crown group. The single *H. robertsi*, all three *H. cretica* and two of three *H. sammuti* samples group on *H. euphorbiae* branches, the third *H. sammuti* on the *H. tithymali* subtree. There are four *H. tithymali* samples (MOR_5356, gESP_8626, YEM_8748, and YEM_8936) on *H. euphorbiae* branches and one *H. euphorbiae* (cESP_1275) within *H. tithymali* (fig. 3). The only well supported branch (87% bootstrap) in the entire lineage tree is a clade of two Tunisian *H. tithymali* (TUN_1835 and TUN_1839) with one from Lanzarote (cLZ_1624), which would be nominally allocated to two different subspecies, *mauretanica* and *tithymali*, respectively.

In the absence of two or more discrete units, it is not adequate to test for gene flow, as the results would be expected to show artifacts, but we performed the analyses using the currently valid species definitions (supplementary table S2 and supplementary fig. S5, Supplementary Material online) and interpret them with caution. MLE and BF comparison of the 19 competing species models found strong statistical support for the species model that merged the five traditional taxonomic species into two, lumping *H. euphorbiae*, *H. cretica*, and *H. sammuti* into one species and *H. robertsi* and *H. tithymali* into a second (MLE = −100,047.7; supplementary table S2, Supplementary Material online). The scenario of the HEC representing one species only was the least likely (last rank), whereas the scenario of the HEC representing the five currently valid species represented an extreme beyond any probability (highest ML value, no rank).

### HEC Species Delineation

The best BF scenario corresponds broadly to a geographic split along the Mediterranean Sea; that is, lumping *H. robertsi* with *H. tithymali*, and *H. cretica* and *H. sammuti* with *H. euphorbiae* (BF species tree, supplementary fig. S5, Supplementary Material online). Although the BF designation into two species (supplementary table S2, Supplementary Material online) coincides with the Structure result, supporting divergence into two groups (implied by the ad hoc Δ*K *=* *2), every single HEC individual is assigned to the blue group with over 80% admixture by Structure (fig. 2*b*). The grouping suggested by the BFD method in supplementary figure S5, Supplementary Material online, *H. robertsi* plus *H. tithymali*, as a second, distinct group, is not reflected in figure 2.

The four-taxon *D*-statistics were used to detect introgression and also to draw ancestry graphs implemented in *TreeMix* (v.1.12; Pickrell and Pritchard 2012; trees with 11 migration events allowed) to identify patterns of divergence and migration within the HEC. High *Z*-score values were observed in many tests among species in the HEC, suggesting potential hybridization and introgression had occurred in the past (supplementary table S3, Supplementary Material online). Both tests are congruent in that the direction of gene flow is from *H. sammuti* to *H. robertsi*, from *H. cretica* to *H. euphorbiae*, from *H. robertsi* to *H. euphorbiae*, and from *H. sammuti* to *H. tithymali* (misleading TreeMix dendrogram is not shown). The strongest signal of ancestral interspecific hybridization, based on four-taxon *D*-statistic tests, was found between the samples of *H. sammuti* and *H. robertsi* and/or between those of *H. sammuti* and *H. euphorbiae* + *H. tithymali* (test 15 in supplementary table S3, Supplementary Material online; 33 cases out of 158). Despite finding significant improvement in fit for up to six events and support for quite substantial introgression in two cases, these results are interpreted as an artifact of unbalanced taxon sampling (due to failure to obtain RADseq data).

Although we cannot provide reliable details of interspecific gene flow due to sample bias (and lack of evidence for discrete groups corresponding to species), overall, the results of our systematic genomic tests of introgression and admixture in the HEC provide statistically rigorous evidence for frequent gene flow causing genomic similarities among the HEC species as currently defined.

## Discussion

### Introgression

The genome-scale SNP data presented here clearly demonstrated a high degree of gene flow and/or introgression within the HEC. It needs to be noted, though, that the sample size of *H. euphorbiae* and *H. tithymali* was much larger than for the other three species (*H. cretica* only 3, *H. robertsi* only 1, *H. sammuti* only 3). This deficiency applies to all analyses (but not to illustrations of divergences). In view of the evidence from conventional genetic data (Bazinet et al. 2013; McCormack et al. 2013), these results can also not be accorded any biological relevance. *Hyles sammuti* is a hybrid taxon based on evidence from mitochondrial sequences and microsatellites. *Hyles euphorbiae* shows incomplete mitochondrial lineage sorting (Mende and Hundsdoerfer 2013; Mende et al. 2016). However, one result may be noteworthy—no support was found for significant introgression between *H. sammuti* and *H. euphorbiae*, because all tests of possible tree topologies involving gene flow between these two species were insignificant. Although unexpected from the geographic scenario, this makes some sense, when compared with mitochondrial data (from Mende and Hundsdoerfer 2013; Mende et al. 2016). In Italy, the nearest potential *H. euphorbiae* source for *H. sammuti*, the mitochondria are not *euphorbiae*, but “italica,” which is a lineage connected to *tithymali* (and *cretica*), but not to *euphorbiae* (Hundsdoerfer, Mende, et al. 2011; Mende et al. 2016).

Our genotypic clustering analyses revealed that the samples of the five HEC species all belong to just a single group (fig. 2*b*) with no internal structure that reflected either geographic origin or IBD (supplementary fig. S1, Supplementary Material online). These results demonstrate unlimited gene flow across the five species as currently defined. The effects are also visible in the SplitsTree networks as reticulations and lack of group formation (supplementary fig. S3, Supplementary Material online), the low *F*_ST_ values (supplementary fig. S2, Supplementary Material online; except for *H. robertsi*, due to missing data and only a single sample), as well as in the PCA where there is overlap of the scatterplots (supplementary fig. S4, Supplementary Material online). Coalescent SVDquartets lineage branching does not coincide with the SVDquartets species tree (fig. 3), revealing ongoing contact in form of gene flow between the species, as well as incomplete lineage sorting. Our study provides clear and multiple evidence for near-random genetic contact among all members of the five currently valid HEC species.

Hybridization has already been recognized in the HEC by Mende et al. (2016), who demonstrated gene flow within glacial refugia and ongoing postglacial gene flow. The mt-sequences of the five HEC species (from Mende et al. 2016) revealed polyphyletic species and subspecies (supplementary fig. S6, Supplementary Material online). With only five steps, the minimal distance of *H. cretica* to either the closest haplotype of the *tithymali* lineage or the italica lineage (supplementary fig. S6, Supplementary Material online) of *H. euphorbiae* is 0.2%. Our data corroborate the hybrid nature of *H. sammuti*, because this species indeed consists of individuals from *H. euphorbiae* and *H. tithymali* lineages (fig. 3). The presence of phylogenetic signal from two a priori species definitions also explains why *H. sammuti* clusters basal to the two large groups in the SVDquartets species tree.

It has been estimated that over 10% of animal species hybridize in nature (Mallet 2005), resulting in introgression (e.g., *Heliconius*, Zhang et al. 2016). These processes complicate species delimitation, often to a degree where species boundaries appear to form a continuum (e.g., Mallet et al. 2007). High levels of hybridization are, however, characteristic of parapatric species pairs and generally take place within narrow contact zones between them. However, the current five species of the HEC do not show such hybrid zones, but are largely allopatric and separated by natural barriers, such as the Mediterranean Sea. Although such natural barriers restrict gene flow, they have not resulted in population differentiation with reproductive isolation in the HEC, probably largely because hawkmoths are strong fliers and long-distance dispersal is common. In such groups of recently evolved species with short intervals between speciation events, shared ancestral polymorphism as well as reticulation due to introgression can obscure data interpretation, making it difficult to discern which taxonomic level to work at. Our results demonstrate that the current five species of the HEC all share the same gene pool.

### Species Boundaries

Species delimitation is often problematic. Complexities arise for two main reasons. First, speciation is a gradual process during which populations slowly become differentiated due to strongly limited or absent gene flow between them. During this process, populations evolve into separate lineages and accumulate specific features that taxonomists then use to delineate species. However, the order and speed at which these features are gained varies between lineages (De Queiroz 1998). Second, the species as a concept has multiple definitions, each using different criteria for determining when species should be considered as being distinct (De Queiroz 2007). Delimitation of allopatric populations, such as those of the HEC, is especially challenging as it can be almost inherently arbitrary (cf., Rosenberg 2004).

Geography and aspects of the external morphology of both the larvae and adults have led a number of authors to describe over two dozen taxa (not counting individual color variations and aberrations) in the HEC in the Western Palearctic region since Linnaeus’ original description of *H. euphorbiae* in 1758 (Kitching and Cadiou 2000). The currently valid taxonomy of Kitching (2019) recognizes five valid species (supplementary table S6, Supplementary Material online; commented in Hundsdoerfer et al. 2005, 2009), but a strict interpretation of larval (Hundsdoerfer, Pittaway, et al. 2011; Hundsdoerfer, Rubinoff, et al. 2009) and adult pattern variability (examples in fig. 3) allows for allocation of individuals into only two groups (plus a range of intermediate forms) based around Central European *Hyles euphorbiaeeuphorbiae* and Canary Islands *Hyles tithymalitithymali*. Two clusters were also corroborated by microsatellite data (Mende et al. 2016). In the present study, Structure assigned all individuals to one group with varying amounts of admixture with a second group (fig. 2*b*). Moreover, the results of the interspecific hybridization test (*D*-statistics) showed significant gene flow within the HEC and no signal due to IBD was detected (supplementary fig. S1, Supplementary Material online). These results explain the genetic similarity among the species, as well as their phylogenetic cohesion.

The SVDquartets coalescence lineage analysis revealed a paraphyletic base consisting of *H. euphorbiae*, and including *H. robertsi*, *H. cretica*, and *H. sammuti*, with *H. tithymali*, as a crown group (fig. 3). The most recent common ancestors of the species as currently defined are all stem nodes (on black stem branches). The grouping of *H. robertsi* is inconsistent depending on the methodological approach used, presumably because only a single sample was included in the study. It pairs with a *H. euphorbiae* sample (TUR_6047) in both the RAxML tree (fig. 2*a*) and the SVDquartets lineage tree (fig. 3), is sister to *H. euphorbiae* in the SVDquartets species tree (fig. 3), but is sister to *H. tithymali* in the BF species tree (supplementary fig. S5, Supplementary Material online). BFD also revealed that the base scenario (the currently valid taxonomy) of five putative species is not supported (supplementary fig. S5, Supplementary Material online). Taken together, the ambiguous placement of the *H. robertsi* sample by different methods clearly demonstrates that this individual is not sufficiently different from all other samples to warrant its status as a separate species.

Using traditional systematic research methodology, our earlier work found incongruence between morphospecies delineation, ecospecies delineation (e.g., using mean habitat temperature; fig. 1), and biospecies delineation (Mende et al. 2016). The polymorphic larvae (Hundsdoerfer, Pittaway, et al. 2011), which show two basic sets of pattern element characters, and differences in larval host plant preferences are examples of traits that are ultimately coded in the DNA. The biological species concept goes back to these roots—that all morphological or ecological variabilities originate from the genome. The overwhelming volume of genome-scale data in this study rounds off the decade-long process of obtaining multiple sources of data for integrative taxonomy (Dayrat 2005) and provides a convincing demonstration that the Western Palearctic HEC constitutes but one gene pool that is a single distinct evolutionary unit in its entirety. Our data describe the HEC as one genealogical lineage, a term that is a common baseline of all species concepts proposed during the last 50 years (De Queiroz 2005). Although secondary properties of lineages such as morphology and ecology can provide evidence for defining subcategories, the other nongenome-scale sources of data applied previously have not yielded an unequivocal concept for doing so in the case of the HEC. The high variability in these characters should be investigated in the future, as a population, not a species, level phenomenon.

The five currently accepted species in the HEC (supplementary fig. S6, Supplementary Material online) appear to overestimate taxonomic diversity and the group is clearly oversplit. The ddRAD data clearly indicate that the individuals of the HEC studied here divide neither into five groups corresponding to the five currently valid species nor into two groups corresponding to two species (that hybridize; see Mende et al. 2016). In a total evidence approach, the genomic data obtained in this study indicate rather convincingly that they all belong to a single species, which then leads to the potentially unpopular step of synonymizing several taxon names. Nevertheless, we consider that this is the correct and justifiable course of action. Consequently, we retain only one name (the oldest) for the entire HEC, *H. euphorbiae*. Furthermore, we choose not to recognize any formally named infraspecific taxa (e.g., former subspecies names, see supplementary fig. S6, Supplementary Material online), even though two clusters (corresponding to *euphorbiae* and *tithymali*) have been found with other sources of data (morphology and geography, e.g., Hundsdoerfer et al. 2009; microsatellites, Mende et al. 2016; summarized in supplementary table S6, Supplementary Material online ).

We therefore propose the following formal taxonomic reclassification of the HEC species rank taxa (names already placed in synonymy and the numerous infrasubspecific names are not listed here; for further information on these, see Kitching and Cadiou 2000):

*Hyles euphorbiae* (Linnaeus, 1758)

*= Deilephila tithymali* Boisduval, 1834 **syn. nov.**

*= Hyles tithymali mauretanica* Staudinger, 1871 **syn. nov.**

*= Deilephila robertsi* Butler, 1880 **syn. nov.**

*= Deilephila peplidis* Christoph, 1894 **syn. nov.**

*= Deilephila mauretanica deserticola* Staudinger, 1901 **syn. nov.**

*= Celerio euphorbiae conspicua* Rothschild and Jordan, 1903 **syn. rev.**

*= Hyles tithymali himyarensis* Meerman, 1988 **syn. nov.**

*= Hyles euphorbiae gecki* de Freina, 1991 **syn. nov.**

= *Hyles robertsi elisabethae* Ebert, 1996 **syn. nov.**

*= Hyles cretica* Eitschberger, Danner and Surholt, 1998 **syn. nov.**

*= Hyles sammuti* Eitschberger, Danner and Surholt, 1998 **syn. nov.**

*= Hyles tithymali gallaeci* Gil-T., Requejo and Estévez, 2011 **syn. nov.**

*= Hyles tithymali phaelipae* Gil-T. and Gil-Uceda, 2012 **syn. nov.**

Several of the former subspecies names (*conspicua*, *deserticola*, *gallaeci*, *gecki*, *himyarensis*, *mauretanica*, *phaelipae*, and *tithymali*; supplementary fig. S6, Supplementary Material online) of the Western Palearctic HEC may be used as informal names for populations reflecting traditional names based on morphological patterns and geography (but not mitochondrial lineages).

This taxonomic revision reflects the view of the late Heimo Harbich (e.g., Harbich 2000), who sadly passed away on January 31, 2017. Harbich consistently ignored the various changes proposed to the valid taxonomy by other authors and adamantly treated the five HEC species in the Western Palearctic as one, albeit with several subspecies (e.g., Harbich 2009). He reported many observations of natural *Hyles* hybrids and also bred numerous hybrid combinations in captivity (e.g., Harbich 1976a, 1984, 2000). Well before the possibilities of genetic analyses, these experiments allowed him to recognize that it appeared remarkable that sympatric species in the genus *Hyles* (then referred to under the invalid genus name, *Celerio*) could be maintained as biological species, rather than fusing into hybrid populations (Harbich 1976b). For more than 50 years, his focus on the HEC yielded an impressive intellectual oeuvre, showing deep understanding of the evolution in this species complex. In this article, and in his memory, we formally implement his species concept.

All methods (morphology, molecular biology, ecology, etc.) have limitations as approaches for delimiting species (cf., Valdecasas et al. 2007). In future, taxonomic oversplitting, that is, the naming of individual variants as distinct specific entities, can be avoided by adopting a more integrative approach, accepting the high degree of complexity in diversity that needs to be studied from multiple and complementary perspectives before a new taxonomic name is proposed (Dayrat 2005). A lack of distinct phylogenetic splits, together with population clustering that lacks structure and does not reflect traditional species names, morphology or geographical origin, indicates an even higher degree of gene flow and a much lower degree of divergence in the HEC than previously thought. The genomic data obtained in this study is so comprehensive that, after nearly 20 years of genetic research on this taxonomically contentious Lepidoptera species complex, the integrative data at hand is finally sufficiently convincing to allow us to undertake the long overdue taxonomic revision of the HEC. With five valid species names, the group had been oversplit. Reducing them to the single taxonomically valid species name, *H. euphorbiae*, also allows former hybridization scenarios for this species complex to be set aside, given that hybridization is per se defined as gene exchange between species.

## Conclusions

Although the adoption of genetic tools (e.g., DNA barcoding, Ratnasingham and Hebert 2013) has frequently revealed morphologically cryptic diversity, the converse, taxonomic oversplitting of species, is perhaps not as rare as might be commonly thought (see Mutanen et al. 2016). Long taxonomic scrutiny may often generate progressively finer-scale taxonomic resolution, a process that eventually leads to a point where boundaries between species are difficult to discern. Such oversplitting of species is most likely to have taken place especially in intensively studied, popular and charismatic organisms, such as the group of hawkmoths studied here. As speciation is usually a slow biological process, delimitation of species is to certain degree inherently arbitrary, especially when allopatric populations are concerned (Mutanen et al. 2012) and when ranges are geographically wide. Genomic tools, such as the RAD-sequencing applied here, have the benefit of allowing quantification of the variables important in species delimitation, including intensity of gene flow between populations and extent of intrapopulation genetic variation at the genome-wide scale. Taxonomically complex cases are many in virtually all groups of organisms. Gaining comprehensive understanding of relationships between populations and hence reaching taxonomic stability will likely often require a genomic insight, and options for that are presently many with new methods continuing to appear. Nevertheless, we encourage authors to interpret their molecular data carefully and critically and not to shy away from taxonomic lumping if this is necessary to reflect biological reality.

## Supplementary Material

Supplementary data are available at *Genome Biology and Evolution* online.

## Supplementary Material

evz113_Supplementary_Data

## Notes

**Data deposition:** This project has been deposited at NCBI Sequence Read Archive under the accession PRJNA352456.
