# Supplementary material for: Genome-wide SNP Data Reveal an Overestimation of Species Diversity in a Group of Hawkmoths
Source: Genome Biol Evol. 2019 May 29;11(8):2136–50. doi: 10.1093/gbe/evz113 (PMC6685492; doi:10.1093/gbe/evz113)
Supplement: evz113_Supplementary_Data [file evz113_supplementary_data.docx]

## Fig. S1. – Isolation by distance (IBD)

Scatterplot from the isolation by distance analyses (IBD; 1,000 randomizations) of the HEC. Dots represent pairwise genetic distances, plotted according the geographic distance between the two individuals. Note: *r*_M_, Mantel test correlation coefficient; *p*, Mantel test significance.


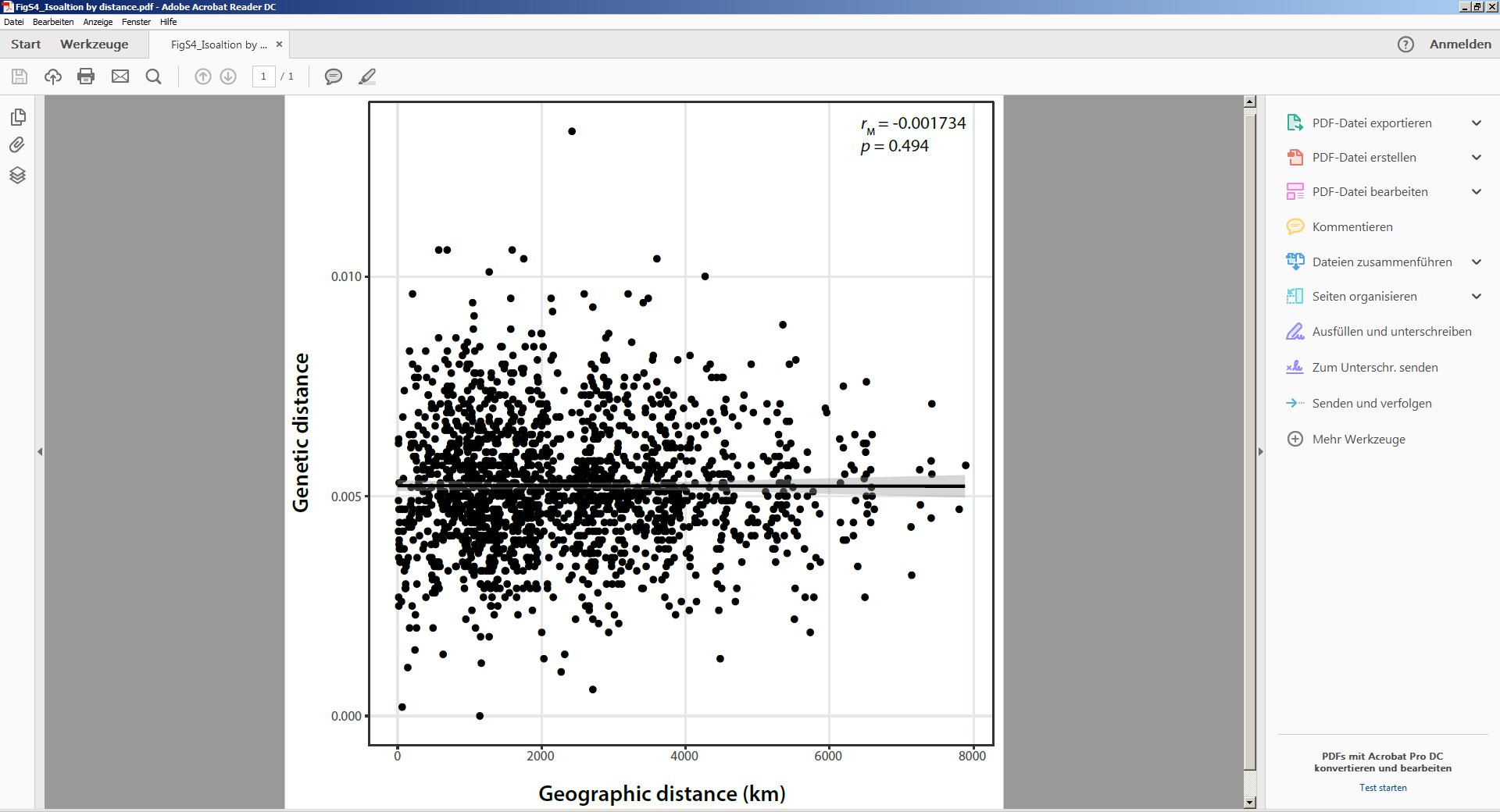


## Fig. S2. – F_ST_ values within the HEC

Pairwise F_ST_ values among all pairs of the HEC. An asterisk denotes significant statistical support from 1,000 permutations (*p* < 0.05).

**
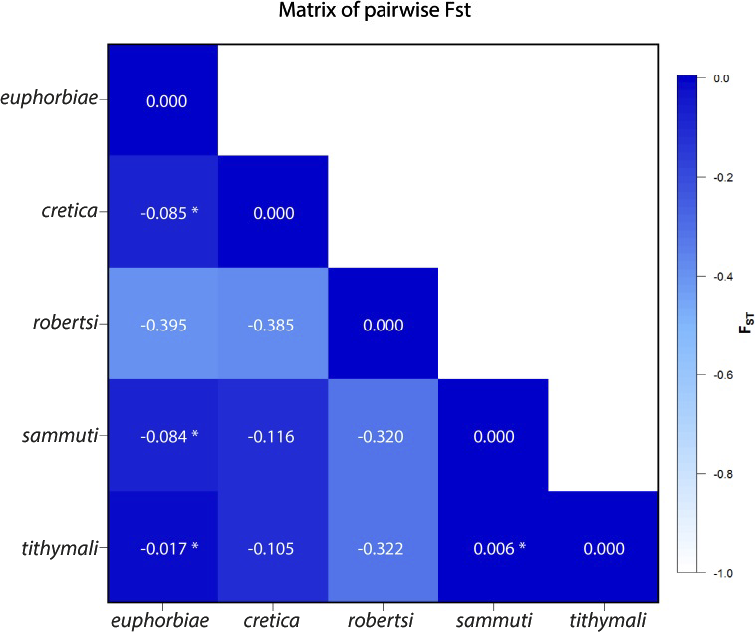
**

## Fig. S3. – SplitsTree Networks of the HEC

Phylogenetic networks of the HEC based on 7,969 SNPs using SplitsTree v.4.14.2 with heterozygous ambiguities averaged and normalized. Boxes in the network represent uncertainty in the phylogeny and are expected if horizontal gene exchange has occurred or incomplete lineage sorting prevails. Bootstrap support values (1,000 replicates) over 75% are shown. Colours follow those in Figure 1 and code currently valid taxonomy. Two different analytical approaches were implemented: a) uncorrected p-distances and b) maximum parsimony.


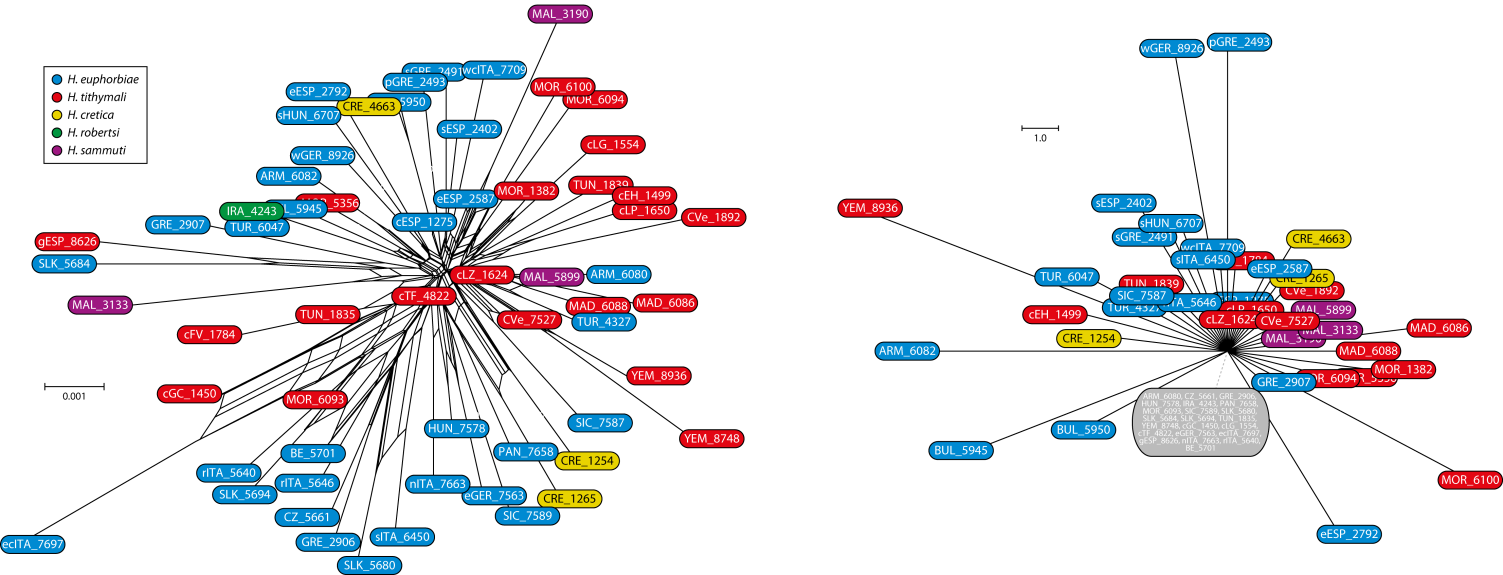


## Fig. S4. – Principal components analysis (PCA)

Scatterplot from principal components analysis (PCA) of the HEC. Dots represent individuals, and colouration and 95% inertia ellipses visualize assignment. Eigenvalues of the first 40 components are shown, with the proportion of total variance scaled on the y-axis. a) PCA results for all five species and separate plots of b) *H. euphorbiae*, c) *H. cretica*, d) *H. tithymali*, e) *H. robertsi*, and f) *H. sammuti*. The number of individuals of each species is shown in parentheses. Colors follow Figs. 1-3 and code currently valid taxonomy.


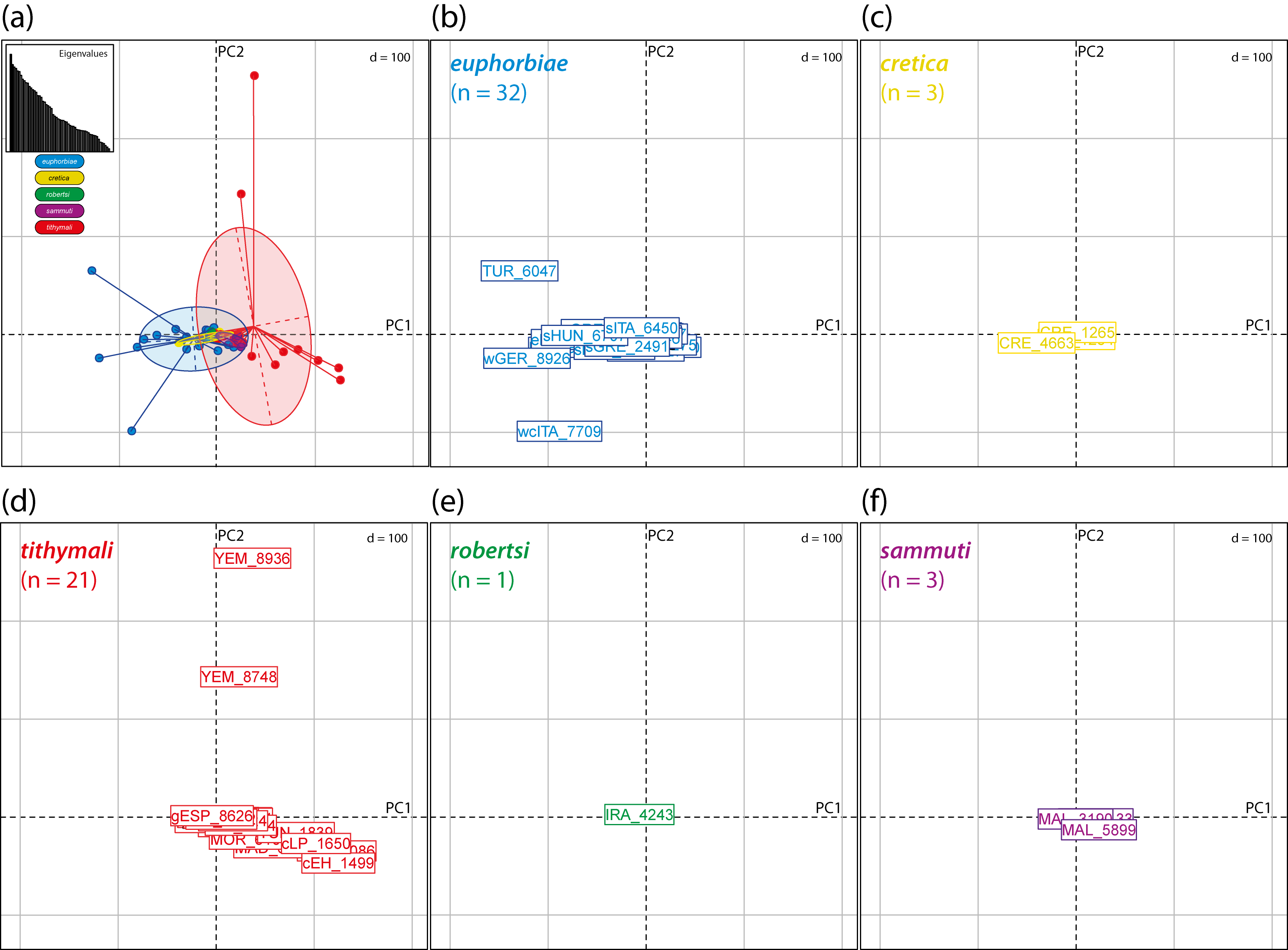


## Fig. S5. – BF Species tree

Bayes factor HEC species tree estimated with 2,465 SNPs (HEC_c85m20 to reduce missing data) based on the best supported BFD*species model (2 sp_rt). An outgroup (*H. dahlii*) was also incorporated into the species tree. Posterior probability is shown near the branch.


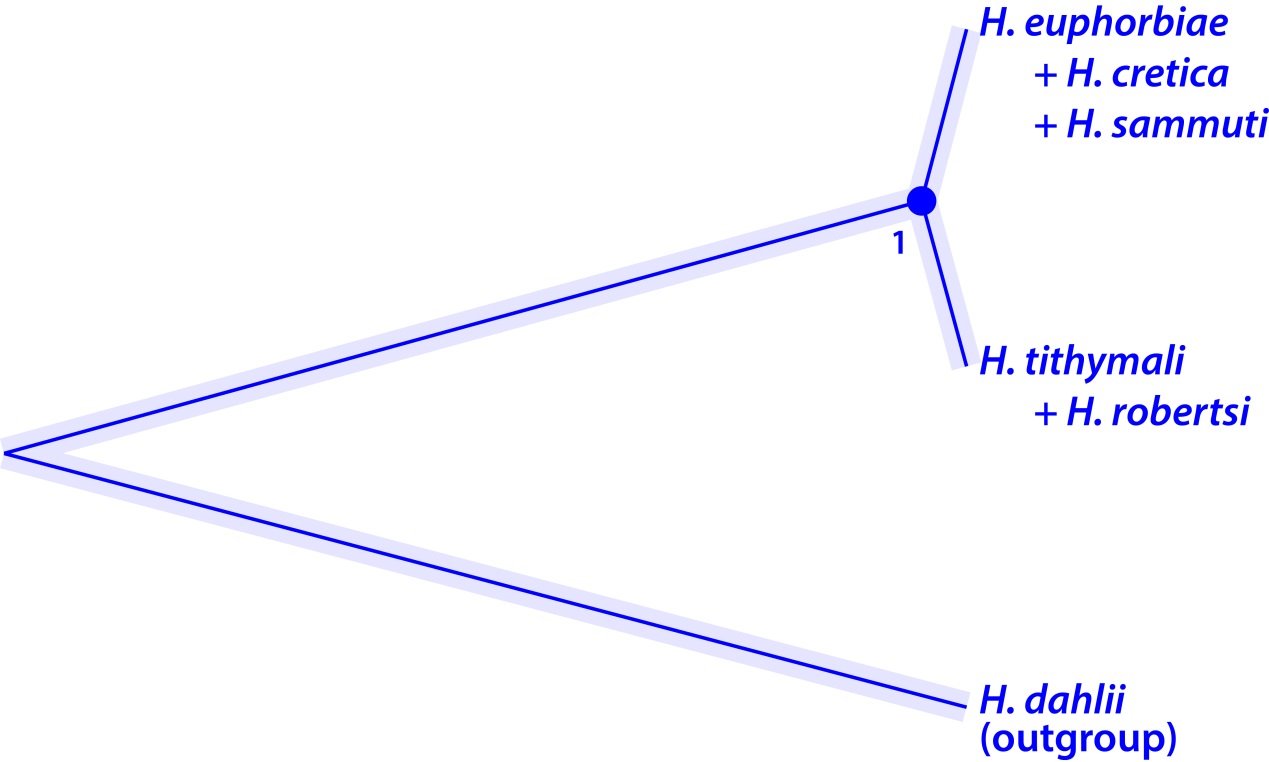


## Fig. S6. – Mitochondrial sequence network of the HEC with species names

Haplotype network of mitochondrial COI/II sequences of the HEC from Mende *et al*. (Mende *et al.* 2016), redrawn to illustrate valid taxonomy. Size of haplotype circles reflects sample size and black nodes represent missing haplotypes. Uncorrected p-distances are given between the seven haplogroups. Sequences of *H. dahlii* are included for comparison.


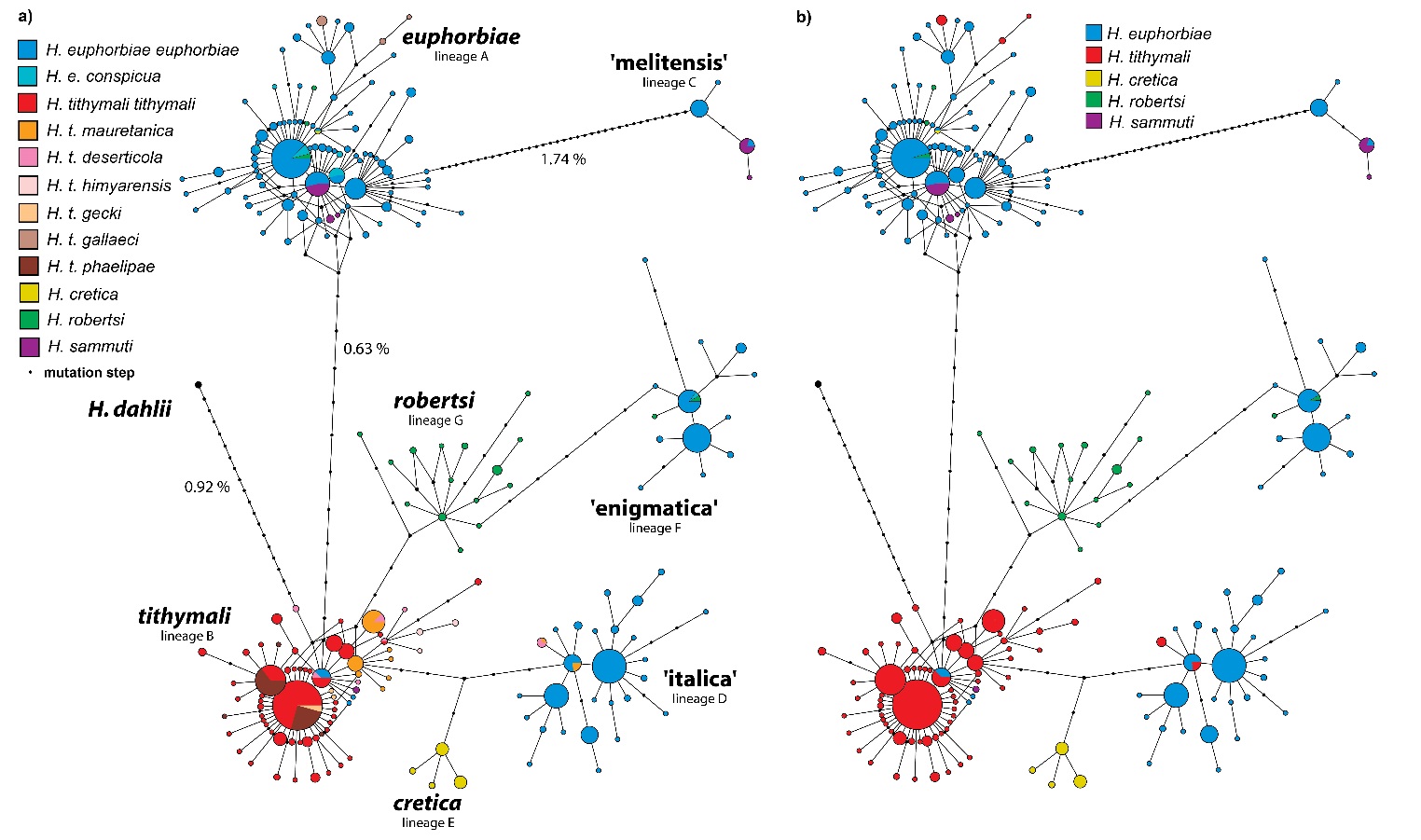


**Table S1 Sequence information with different parameters**

Sequence information in the ddRAD data matrices (n=62) generated with different parameters of clustering threshold (*c*) and minimum number of individuals per locus (*m*) values. The data matrices shown in bold type were used for analyses.

| **Matrix** | **Loci** | **Unlinked SNPs** | **Consensus sequences (bp)** | **VAR** | **PIS** | **Missing (%)** |
| --- | --- | --- | --- | --- | --- | --- |
| HEC_*c*80*m*20 | 2,484 | 2,471 | 480,657 | 32,053 | 17,127 | 55.9 |
| **HEC_*c*85*m*6** | 11,276 | 10,093 | 2,174,137 | 114,463 | 56,033 | 86.2 |
| HEC_*c*85*m*10 | 6,619 | 6,402 | 1,278,801 | 76,379 | 40,405 | 70.0 |
| HEC_*c*85*m*15 | 3,975 | 3,939 | 767,422 | 47,417 | 25,887 | 62.4 |
| HEC_*c*85*m*20 | 2,477 | 2,465 | 477,342 | 28,673 | 15,487 | 47.7 |
| HEC_*c*85*m*25 | 1,478 | 1,473 | 284,347 | 15,917 | 8,496 | 49.7 |
| **HEC_*c*85*m*30** | 837 | 835 | 160,778 | 7,969 | 4,005 | 44.0 |
| HEC_*c*90*m*20 | 2,263 | 2,252 | 434,561 | 22,949 | 12,047 | 55.9 |

## Table S2 Species delimitation hypotheses of the HEC

Different species delimitation models for the group evaluated with the BFD* method and their results. Each row indicates a different species delimitation model and *H. dahlii* was included in all tests as outgroup. The best delimitation scenario is shown in bold. Abbreviations refer to current species definitions: c, *H. cretica*; e, *H. euphorbiae*; r, *H. robertsi*; s, *H. sammuti*; t, *H. tithymali*, sp, species.

| **Scenarios** | **Description** | **Species number** | **MLE** | **BF** | **Rank** |
| --- | --- | --- | --- | --- | --- |
| Lump all | c+e+r+s+t | 1 | -100151.3 | -139.7 | 18 |
| 2 sp_ce | c+e / r+s+t | 2 | -100083.6 | -275.0 | 16 |
| 2 sp_cr | c+r / e+s+t | 2 | -100051.6 | -339.0 | 4 |
| 2 sp_cs | c+s / e+r+t | 2 | -100067.6 | -307.0 | 14 |
| 2 sp_ct | c+t / e+r+s | 2 | -100062.6 | -317.1 | 12 |
| 2 sp_er | e+r / c+s+t | 2 | -100060.8 | -320.8 | 11 |
| 2 sp_es | e+s / c+r+t | 2 | -100063.9 | -314.4 | 13 |
| 2 sp_et | e+t / c+r+s | 2 | -100053.1 | -336.0 | 5 |
| 2 sp_rs | r+s / c+e+t | 2 | -100050.8 | -340.6 | 3 |
| **2 sp_rt** | **r+t / c+e+s** | **2** | **-100047.7** | **-346.8** | **1** |
| 2 sp_st | s+t / c+e+r | 2 | -100057.5 | -327.2 | 9 |
| 2 sp_c | c / e+r+s+t | 2 | -100050.1 | -342.1 | 2 |
| 2 sp_e | e / c+r+s+t | 2 | -100059.2 | -323.9 | 10 |
| 2 sp_r | r / c+e+s+t | 2 | -100055.6 | -331.0 | 7 |
| 2 sp_s | s / c+e+r+t | 2 | -100055.8 | -330.6 | 8 |
| 2 sp_t | t / c+e+r+s | 2 | -100054.1 | -334.1 | 6 |
| 3 sp_cer | c+e+r / s / t | 3 | -100067.7 | -306.8 | 15 |
| 4 sp_ce | c+e / r / s / t | 4 | -100104.9 | -232.4 | 17 |
| Trad. taxon | c / e / r / s /t | 5 | -100221.1 | NA | NA |

## Table S3 Results of the four-taxon D-statistic tests for admixture

The values show significant replicates for introgression in the HEC. Each test was repeated over all possible four-sample replicates (n), with a range of Z-scores reported, and the number of significant replicates shown (nSig). P1, P2, and P3 are ingroups that refer to current species definitions: c, *H. cretica*; e, *H. euphorbiae*; r, *H. robertsi*; s, *H. sammuti*; t, *H. tithymali*. The outgroup, not shown in the table, consists of two individuals of *H. dahlii*.

| **Test** | **P1** | **P2** | **P3** | **Range Z** | **nSig/n** | **nSig/n (%)** |
| --- | --- | --- | --- | --- | --- | --- |
| 1 | c | c | e | (0.0 – 31.6) | 9/95 | 9.5 |
| 2 | e | e | c | (0.0 – 31.6) | 85/1487 | 5.7 |
| 3 | e | r | s | (0.0 – 19.1) | 16/95 | 16.8 |
| 4 | e | s | r | (0.0 – 8.5) | 6/95 | 6.3 |
| 5 | e | (cr) | s | (0.0 – 18.3) | 20/383 | 5.2 |
| 6 | r | s | (et) | (0.0 – 9.0) | 10/158 | 6.3 |
| 7 | s | c | e | (0.0 – 37.6) | 16/287 | 5.6 |
| 8 | s | r | e | (0.0 – 9.3) | 7/95 | 7.4 |
| 9 | s | (cr) | e | (0.0 – 37.7) | 22/383 | 5.7 |
| 10 | t | t | r | (0.0 – 14.2) | 12/209 | 5.7 |
| 11 | (ec) | r | s | (0.0 – 31.6) | 17/104 | 16.3 |
| 12 | (ec) | s | r | (0.0 – 10.2) | 7/104 | 6.7 |
| 13 | (er) | t | s | (0.0 – 31.6) | 104/2078 | 5.0 |
| 14 | (et) | c | r | (0.0 – 9.6) | 10/158 | 6.3 |
| 15 | (et) | r | s | (0.0 – 31.6) | 33/158 | 20.9 |
| 16 | (et) | s | r | (0.0 – 9.3) | 8/158 | 5.1 |
| 17 | c | r | (et) | (0.0 – 8.4) | 3/62 | 4.8 |
| 18 | c | r | e | (0.0 – 9.3) | 3/158 | 1.9 |
| 19 | c | s | (et) | (0.0 – 9.2) | 3/95 | 3.2 |
| 20 | c | (rs) | e | (0.0 – 38.1) | 17/476 | 3.6 |
| 21 | e | c | r | (0.0 – 37.8) | 18/383 | 4.7 |
| 22 | e | c | s | (0.0 – 9.5) | 4/95 | 4.2 |
| 23 | e | c | t | (0.0 – 13.5) | 6/287 | 2.1 |
| 24 | e | e | r | (0.0 – 22.9) | 51/2015 | 2.5 |
| 25 | e | e | s | (0.0 – 9.4) | 18/495 | 3.6 |
| 26 | e | r | c | (0.0 – 31.6) | 60/1487 | 4.0 |
| 27 | e | r | t | (0.0 – 9.2) | 4/95 | 4.2 |
| 28 | e | s | c | (0.0 – 18.3) | 25/671 | 3.7 |
| 29 | e | t | (cr) | (0.0 – 27.3) | 11/287 | 3.8 |
| 30 | e | t | (crs) | (0.0 – 40.0) | 95/2687 | 3.5 |
| 31 | e | t | (cs) | (0.0 – 39.3) | 171/4703 | 3.6 |
| 32 | e | t | (rs) | (0.0 – 38.1) | 162/4031 | 4.0 |
| 33 | e | t | c | (0.0 – 22.4) | 98/2687 | 3.6 |
| 34 | e | t | r | (0.0 – 39.0) | 89/2015 | 4.4 |
| 35 | e | t | s | (0.0 – 22.4) | 16/671 | 2.4 |
| 36 | e | (crs) | t | (0.0 – 31.6) | 88/2015 | 4.4 |
| 37 | e | (cs) | r | (0.0 – 28.5) | 130/4703 | 2.8 |
| 38 | e | (rs) | c | (0.0 – 9.1) | 7/191 | 3.7 |
| 39 | r | (cs) | e | (0.0 – 27.2) | 15/383 | 3.9 |
| 40 | s | s | e | (0.0 – 9.1) | 9/191 | 4.7 |
| 41 | s | s | t | (0.1 – 7.9) | 4/95 | 4.2 |
| 42 | t | c | e | (0.0 – 5.3) | 3/62 | 4.8 |
| 43 | t | r | e | (0.0 – 11.7) | 2/107 | 1.9 |
| 44 | t | s | e | (0.0 – 22.4) | 27/671 | 4.0 |
| 45 | t | t | c | (0.0 – 22.4) | 61/2015 | 3.0 |
| 46 | t | t | e | (0.0 – 31.6) | 12/629 | 1.9 |
| 47 | t | t | s | (0.0 – 31.6) | 152/6719 | 2.3 |
| 48 | t | t | (er) | (0.0 – 22.4) | 29/629 | 4.6 |
| 49 | t | (cr) | e | (0.0 – 31.6) | 163/6929 | 2.4 |
| 50 | t | (cs) | e | (0.0 – 31.6) | 100/2687 | 3.7 |
| 51 | t | (rs) | e | (0.0 – 31.6) | 125/4031 | 3.1 |
| 52 | (cr) | s | (et) | (0.0 – 31.6) | 83/2687 | 3.1 |
| 53 | (cs) | r | (et) | (0.0 – 37.2) | 24/635 | 3.8 |
| 54 | (ec) | r | t | (0.0 – 10.1) | 12/317 | 3.8 |
| 55 | (ec) | t | (rs) | (0.0 – 18.3) | 27/734 | 3.7 |
| 56 | (ec) | t | r | (0.0 – 31.6) | 106/2939 | 3.6 |
| 57 | (ec) | t | s | (0.0 – 31.6) | 23/734 | 3.1 |
| 58 | (ecr) | s | t | (0.0 – 31.6) | 89/2204 | 4.0 |
| 59 | (ecs) | r | t | (0.0 – 31.6) | 76/2267 | 3.4 |
| 60 | (er) | c | s | (0.0 – 31.6) | 29/797 | 3.6 |
| 61 | (er) | c | t | (0.0 – 13.1) | 7/296 | 2.4 |
| 62 | (er) | s | c | (0.0 – 31.6) | 50/2078 | 2.4 |
| 63 | (er) | s | t | (0.0 – 27.2) | 11/296 | 3.7 |
| 64 | (er) | t | (cs) | (0.0 – 22.4) | 67/2078 | 3.2 |
| 65 | (er) | t | c | (0.0 – 39.8) | 179/4157 | 4.3 |
| 66 | (erc) | t | s | (0.0 – 40.1) | 88/2078 | 4.2 |
| 67 | (ers) | c | t | (0.0 – 22.4) | 108/2267 | 4.8 |
| 68 | (ers) | t | c | (0.0 – 31.6) | 56/2267 | 2.5 |
| 69 | (es) | c | r | (0.0 – 39.7) | 80/2267 | 3.5 |
| 70 | (es) | c | t | (0.0 – 9.4) | 5/104 | 4.8 |
| 71 | (es) | r | c | (0.0 – 31.6) | 57/2204 | 2.6 |
| 72 | (es) | r | t | (0.0 – 7.1) | 4/104 | 3.8 |
| 73 | (es) | t | (cr) | (0.0 – 14.2) | 28/734 | 3.8 |
| 74 | (es) | t | c | (0.0 – 39.8) | 94/2939 | 3.2 |
| 75 | (es) | t | r | (0.0 – 38.9) | 84/2204 | 3.8 |
| 76 | (esc) | t | r | (0.0 – 12.0) | 18/734 | 2.5 |
| 77 | (et) | c | s | (0.0 – 10.1) | 24/797 | 3.0 |
| 78 | (et) | r | c | (0.0 – 12.8) | 12/476 | 2.5 |
| 79 | (et) | s | c | (0.0 – 7.0) | 6/158 | 3.8 |
| 80 | c | c | t | (0.0 – 26.4) | 12/476 | 2.5 |

## Table S4 Summary of the individual-level ddRAD data

Country codes in Sample ID correspond to population definitions in Figure 1.

| **Species** | **Sample ID** | **Total reads (x10^6^)** | **Retained reads (%)** | **Clusters at 85%^a^** | **Retained loci^b^** | **Coverage^c^** | **Recovered loci** |
| --- | --- | --- | --- | --- | --- | --- | --- |
| *H. dahlii* | 2391_Hdahl | 0.24 | 73.4 | 24395 | 6115 | 15.0 | 2911 |
| *H. dahlii* | 4660_Hdahl | 0.36 | 75.3 | 19315 | 5459 | 15.4 | 2516 |
| *H. cretica* | CRE_1254 | 0.35 | 76.2 | 11243 | 5224 | 24.3 | 1344 |
| *H. cretica* | CRE_1265 | 0.15 | 71.0 | 7941 | 2077 | 18.9 | 442 |
| *H. cretica* | CRE_4663 | 0.20 | 71.7 | 11230 | 4149 | 15.3 | 1358 |
| *H. euphorbiae* | ARM_6080 | 0.35 | 71.9 | 14323 | 6217 | 15.7 | 1681 |
| *H. euphorbiae* | ARM_6082 | 0.24 | 67.8 | 14363 | 6876 | 14.1 | 1723 |
| *H. euphorbiae* | BE_5701 | 0.02 | 68.2 | 1635 | 537 | 15.7 | 170 |
| *H. euphorbiae* | BUL_5945 | 0.23 | 73.8 | 14143 | 5731 | 16.2 | 1616 |
| *H. euphorbiae* | BUL_5950 | 0.37 | 73.9 | 15046 | 7683 | 21.0 | 1902 |
| *H. euphorbiae* | cESP_1275 | 0.34 | 75.9 | 13527 | 5472 | 16.9 | 1694 |
| *H. euphorbiae* | CZ_5661 | 0.19 | 76.7 | 2606 | 1069 | 33.6 | 356 |
| *H. euphorbiae* | ecITA_7697 | 0.17 | 73.4 | 9068 | 2230 | 18.0 | 146 |
| *H. euphorbiae* | eESP_2587 | 0.07 | 66.8 | 8841 | 2645 | 10.3 | 913 |
| *H. euphorbiae* | eESP_2792 | 0.30 | 76.1 | 13321 | 7404 | 22.3 | 1867 |
| *H. euphorbiae* | eGER_7563 | 0.11 | 79.0 | 5086 | 1369 | 21.8 | 414 |
| *H. euphorbiae* | GRE_2906 | 0.05 | 70.5 | 2631 | 893 | 20.1 | 259 |
| *H. euphorbiae* | GRE_2907 | 0.12 | 70.6 | 4207 | 1464 | 17.2 | 400 |
| *H. euphorbiae* | HUN_7578 | 0.22 | 73.2 | 10147 | 3010 | 8.5 | 885 |
| *H. euphorbiae* | nITA_7663 | 0.09 | 69.0 | 9419 | 2357 | 11.5 | 719 |
| *H. euphorbiae* | PAN_7658 | 0.16 | 74.1 | 6030 | 1596 | 31.8 | 453 |
| *H. euphorbiae* | pGRE_2493 | 0.27 | 75.8 | 11283 | 6676 | 20.1 | 1704 |
| *H. euphorbiae* | rITA_5640 | 0.22 | 75.3 | 3966 | 995 | 35.8 | 346 |
| *H. euphorbiae* | rITA_5646 | 0.09 | 69.1 | 5600 | 2078 | 13.5 | 710 |
| *H. euphorbiae* | sESP_2402 | 0.68 | 75.9 | 19056 | 9203 | 29.8 | 1941 |
| *H. euphorbiae* | sGRE_2491 | 0.17 | 73.7 | 10904 | 5065 | 16.3 | 1420 |
| *H. euphorbiae* | sHUN_6707 | 0.19 | 75.6 | 12642 | 6691 | 14.5 | 1915 |
| *H. euphorbiae* | SIC_7587 | 0.22 | 74.1 | 12519 | 4809 | 16.0 | 1024 |
| *H. euphorbiae* | SIC_7589 | 0.22 | 74.3 | 12294 | 3232 | 23.7 | 560 |
| *H. euphorbiae* | sITA_6450 | 0.31 | 74.9 | 4628 | 2332 | 29.1 | 787 |
| *H. euphorbiae* | SLK_5680 | 0.03 | 64.8 | 3294 | 1007 | 11.8 | 312 |
| *H. euphorbiae* | SLK_5684 | 0.04 | 74.8 | 3048 | 1036 | 14.0 | 324 |
| *H. euphorbiae* | SLK_5694 | 0.07 | 75.1 | 2575 | 799 | 18.4 | 265 |
| *H. euphorbiae* | TUR_4327 | 0.15 | 76.1 | 12238 | 5506 | 12.6 | 1542 |
| *H. euphorbiae* | TUR_6047 | 0.50 | 74.1 | 20111 | 10550 | 22.8 | 1940 |
| *H. euphorbiae* | wcITA_7709 | 0.49 | 79.1 | 17722 | 10676 | 24.0 | 1911 |
| *H. euphorbiae* | wGER_8926 | 0.47 | 78.0 | 18465 | 9832 | 26.2 | 1994 |
| *H. robertsi* | IRA_4243 | 0.04 | 67.3 | 5326 | 1094 | 12.0 | 385 |
| *H. sammuti* | MAL_3133 | 0.21 | 77.3 | 6854 | 3930 | 18.4 | 1066 |
| *H. sammuti* | MAL_3190 | 0.04 | 72.9 | 3582 | 1895 | 10.2 | 564 |
| *H. sammuti* | MAL_5899 | 0.30 | 73.2 | 15359 | 6509 | 14.5 | 1733 |
| *H. tithymali* | cEH_1499 | 0.50 | 76.6 | 15239 | 8837 | 31.2 | 2035 |
| *H. tithymali* | cFV_1784 | 0.18 | 73.0 | 6427 | 2776 | 18.3 | 799 |
| *H. tithymali* | cGC_1450 | 0.33 | 73.2 | 4246 | 792 | 41.1 | 177 |
| *H. tithymali* | cLG_1554 | 0.44 | 74.5 | 8664 | 2644 | 41.3 | 651 |
| *H. tithymali* | cLP_1650 | 0.32 | 73.7 | 13351 | 7400 | 22.7 | 1929 |
| *H. tithymali* | cLZ_1624 | 0.39 | 68.5 | 14355 | 4861 | 22.5 | 793 |
| *H. tithymali* | cTF_4822 | 0.11 | 66.7 | 6449 | 1238 | 25.4 | 257 |
| *H. tithymali* | CVe_1892 | 0.21 | 76.2 | 10644 | 5698 | 19.0 | 1518 |
| *H. tithymali* | CVe_7527 | 0.19 | 76.0 | 17537 | 6365 | 12.8 | 1745 |
| *H. tithymali* | gESP_8626 | 0.34 | 77.9 | 3911 | 1245 | 56.9 | 294 |
| *H. tithymali* | MAD_6086 | 0.23 | 74.5 | 13802 | 7504 | 16.6 | 1906 |
| *H. tithymali* | MAD_6088 | 0.27 | 72.0 | 16488 | 7016 | 16.2 | 1823 |
| *H. tithymali* | MOR_1382 | 0.18 | 68.3 | 12775 | 4012 | 15.1 | 1043 |
| *H. tithymali* | MOR_5356 | 0.14 | 64.3 | 12853 | 4859 | 9.8 | 1439 |
| *H. tithymali* | MOR_6093 | 0.41 | 70.1 | 10351 | 4822 | 39.3 | 220 |
| *H. tithymali* | MOR_6094 | 0.26 | 75.3 | 10671 | 4266 | 21.4 | 1098 |
| *H. tithymali* | MOR_6100 | 0.46 | 72.1 | 13983 | 7050 | 18.3 | 1609 |
| *H. tithymali* | TUN_1835 | 0.48 | 79.0 | 4771 | 845 | 176.3 | 279 |
| *H. tithymali* | TUN_1839 | 0.67 | 75.5 | 15336 | 9396 | 38.3 | 2011 |
| *H. tithymali* | YEM_8748 | 0.35 | 74.5 | 8878 | 3952 | 28.7 | 1150 |
| *H. tithymali* | YEM_8936 | 0.22 | 74.4 | 15553 | 6848 | 14.7 | 1815 |
|  |  | **0.25** | **73.3** | **10585** | **4450** | **23.4** | **1142** |

Note: values in the last line are averages.

^a^Clusters that passed filtering for 3x minimum coverage.

^b^Loci retained after passing coverage and paralog filters.

^c^Mean depth of loci.

## Table S5 The summary of Evanno output.

The best supported model is shown in bold type.

| **K** | **Replicates** | **Mean LnP (K)** | **Stdev LnP (K)** | **Ln’(K)** | **\|Ln''(K)\|** | **∆K** |
| --- | --- | --- | --- | --- | --- | --- |
| 1 | 10 | -21528.00 | 2.59 | NA | NA | NA |
| **2** | **10** | **-20777.65** | **39.69** | **750.35** | **325.37** | **8.20** |
| 3 | 10 | -20352.67 | 122.03 | 424.98 | 115.21 | 0.94 |
| 4 | 10 | -20042.90 | 456.83 | 309.77 | 104.43 | 0.23 |
| 5 | 10 | -19628.70 | 215.45 | 414.20 | NA | NA |

## Table S6 Summary of additional sources of data that subdivide the HEC into populations.

Percentages reported in the columns reflect group formation based on geography, the microsatellite and mitochondrial data from Mende *et al.* (Mende et al. 2016). The names of the populations (and some mt-lineages) are not taxonomic names and are thus formatted without italics.

| **Traditional, former Taxa** | **Geography** | **Microsatellite Assignment** | **Mitochondrial Lineage-Assignment** | **Population** |
| --- | --- | --- | --- | --- |
| *H. euphorbiae* | Central Europe | *euphorbiae* (>80%) | *euphorbiae* (~70%, enigmatica ~25%) | euphorbiae |
| *H. euphorbiae* | Italy, Sicily | *euphorbiae* (>80%) | italica (> 80%) | grentzenbergi |
| *H. e. conspicua* | Eastern Europe | *euphorbiae* | *euphorbiae,* enigmatica | conspicua |
| *H. t. tithymali* | Canaries, Cape Verde | *tithymali* | *tithymali* | tithymali |
| *H. tithymali phaelipae* | Western Canaries | *tithymali* | *tithymali* | phaelipae |
| *H. tithymali gecki* | Azores | *tithymali* | *tithymali* | gecki |
| *H. tithymali mauretanica* | Morocco | *tithymali* (>60%  *euphorbiae* <40%) | *tithymali* | mauretanica |
| *H. tithymali deserticola* | Tunisia | *tithymali* (*>*80%) | *tithymali* (> 80%) | deserticola |
| *H. tithymali himyarensis* | Yemen | *euphorbiae* | *tithymali* | himyarensis |
| *H. tithymali gallaeci* | Galicia | *euphorbiae* | *euphorbiae* | gallaeci |
| *H. sammuti* | Malta | *tithymali* ~60%,  *euphorbiae ~*40% | *euphorbiae* (>60%), *tithymali* (~15%), melitensis (~30%) | sammuti |
| *H. robertsi* | Iran | *euphorbiae* (>80%) | *robertsi* (~70%) | robertsi |
| *H. cretica* | Crete | *euphorbiae* (>80%) | *cretica* | cretica |
